# Evaluating the impact of malaria rapid diagnostic tests on patient-important outcomes in sub-Saharan Africa: a systematic review of study methods to guide effective implementation

**DOI:** 10.1136/bmjopen-2023-077361

**Published:** 2024-09-10

**Authors:** Jenifer Akoth Otieno, Lisa Malesi Were, Caleb Kimutai Sagam, Simon Kariuki, Eleanor Ochodo

**Affiliations:** 1 Centre for Global Health Research, Kenya Medical Research Institute, Kisumu, Kenya; 2 Centre for Evidence-Based Health Care, Division of Epidemiology and Biostatistics, Faculty of Medicine and Health Sciences, Stellenbosch University, Cape Town, South Africa

**Keywords:** Malaria, INFECTIOUS DISEASES, Tropical medicine

## Abstract

**ABSTRACT:**

**Objective:**

To perform critical methodological assessments on designs, outcomes, quality and implementation limitations of studies evaluating the impact of malaria rapid diagnostic tests (mRDTs) on patient-important outcomes in sub-Saharan Africa.

**Design:**

A systematic review of study methods.

**Data sources:**

MEDLINE, EMBASE, Cochrane Library, African Index Medicus and clinical trial registries were searched up to May 2022.

**Eligibility criteria:**

Primary quantitative studies that compared mRDTs to alternative diagnostic tests for malaria on patient-important outcomes within sub-Sahara Africa.

**Data extraction and synthesis:**

Studies were sought by an information specialist and two independent reviewers screened for eligible records and extracted data using a predesigned form using Covidence. Methodological quality was assessed using the National Institutes of Health tools. Descriptive statistics and thematic analysis guided by the Supporting the Use of Research Evidence framework were used for analysis. Findings were presented narratively, graphically and by quality ratings.

**Results:**

Our search yielded 4717 studies, of which we included 24 quantitative studies; (15, 62.5%) experimental, (5, 20.8%) quasi-experimental and (4, 16.7%) observational studies. Most studies (17, 70.8%) were conducted within government-owned facilities. Of the 24 included studies, (21, 87.5%) measured the therapeutic impact of mRDTs. Prescription patterns were the most reported outcome (20, 83.3%). Only (13, 54.2%) of all studies reported statistically significant findings, in which (11, 45.8%) demonstrated mRDTs’ potential to reduce over-prescription of antimalarials. Most studies (17, 70.8%) were of good methodological quality; however, reporting sample size justification needs improvement. Implementation limitations reported were mostly about health system constraints, the unacceptability of the test by the patients and low trust among health providers.

**Conclusion:**

Impact evaluations of mRDTs in sub-Saharan Africa are mostly randomised trials measuring mRDTs’ effect on therapeutic outcomes in real-life settings. Though their methodological quality remains good, process evaluations can be incorporated to assess how contextual concerns influence their interpretation and implementation.

**PROSPERO registration number:**

CRD42018083816.

**STRENGTHS AND LIMITATIONS OF THIS STUDY**

- We conducted a robust literature search to get a recent representative sample of articles to assess the methodology.
- In addition to the methodology of studies, we evaluated the implementation challenges that limit the effect of the tests.
- We only included studies published in English which might have limited the generalisability of study findings, but we believe this is a representative sample to investigate the methods used to assess the impact of malaria rapid diagnostic tests.

## Introduction

 The malaria burden remains high in sub-Saharan Africa despite several interventions deployed to control.^1^ Interventions include but are not limited to the adoption of parasitological confirmation of malaria infection using malaria rapid diagnostic tests (mRDTs) and effective treatment using artemisinin-based combination therapies.^2 3^ In 2021, there were 247 million cases of malaria reported globally, an increase of 2 million cases from 245 million cases reported in 2020.^4^ This estimated increase in 2021 was mainly reported in sub-Saharan Africa.^4^ Of all global malaria cases in 2021, 48.1% were reported in sub-Saharan Africa—Nigeria (26.6%), the Democratic Republic of the Congo (DRC) (12.3%), Uganda (5.1%) and Mozambique (4.1%).^4^,^6^ Similarly, 51.9% of the worldwide malaria deaths were reported in sub-Saharan African—Nigeria (31.3%), the DRC (12.6%), the United Republic of Tanzania (4.1%) and Niger (3.9%).^4^,^6^

Following the 2010 WHO’s policy on recommending parasitological diagnosis of malaria before treatment, the availability and access to mRDTs have significantly increased.^7^ For instance, globally, manufacturers sold 3.5 billion mRDTs for malaria between 2010 and 2021, with almost 82% of these sales being in sub-Saharan African countries.^4^ In the same period, National Malaria Control Programmes distributed 2.4 billion mRDTs globally, with 88% of the distribution being in sub-Saharan Africa.^4^ This demonstrates impressive strides in access to diagnostic services in the public sector but does not effectively reveal the extent of test access in the private and retail sectors. Published literature indicates that over-the-counter (OTC) malaria medications or treatment in private retail drug stores are often the first point of care for fever or acute illness in African adults and children.^7^,^9^ Using mRDTs in private drug outlets remains low, leading to overprescribing antimalarials. Increased access to mRDTs may minimise the overuse of OTC medicines to treat malaria.

Universal access to malaria diagnosis using quality-assured diagnostic tests is a crucial pillar of the WHO’s Global Technical Strategy (GTS) for malaria control and elimination.^4 10 11^ Assessing the role of mRDTs in achieving the GTS goals and their impact on patient-important outcomes is essential in effectively guiding their future evaluation and programmatic scale-up.^12^ Rapidly and accurately identifying those with the disease in a population is crucial to administering timely and appropriate treatment. It plays a key role in effective disease management, control and surveillance.

Impact evaluations determine if and how well a programme or intervention works. If impact evaluations are well conducted, they are expected to inform the scale-up of interventions such as mRDTs, including the cost associated with the implementation. Recent secondary research (systematic reviews on the impact of mRDTs on patient-important outcomes)^13^ is only based on assessing mRDTs’ effect and does not consider how well the individual studies were conducted. Odaga *et al* conducted a Cochrane review comparing mRDTs to clinical diagnosis. They included seven trials where mRDTs substantially reduced antimalarial prescription and improved patient health outcomes. However, they did not assess the contextual factors that influence the effective implementation of the studies. There is a need to access the methodological implementation of studies that evaluate the impact of mRDTs. To our knowledge, no study has investigated the implementation methods of studies evaluating the impact of mRDTs.

We aimed to perform critical methodological assessments on the designs, outcomes, quality and implementation limitations of studies that evaluate the impact of mRDTs compared with other malaria diagnostic tests on patient-important outcomes among persons suspected of malaria in sub-Saharan Africa. We defined patient-important outcomes as; characteristics valued by patients which directly reflect how they feel, function or survive (direct downstream health outcomes such as morbidity, mortality and quality of life) and those that lie on the causal pathway through which a test can affect a patient’s health, and thus predict patient health outcomes (indirect upstream outcomes such as time to diagnosis, prescription patterns of antimalarials and antimicrobials, patient adherence).^14^

## Methods

We prepared this manuscript according to the reporting guideline: Preferred Reporting Items for Systematic Reviews and Meta-Analyses (PRISMA-2020)^15^ (online supplemental files 1; 2). The protocol is registered with the International Prospective Register of Systematic Reviews and was last updated in June 2022. The protocol is also available as a preprint in the Open Science Network repositories.^12^

### Patient and public involvement

None.

### Criteria for including studies in this review

#### Study designs

We included primary quantitative studies published in English. We included observational and experimental studies in either controlled or uncontrolled settings. We did not limit trials to the unit of randomisation (individual or cluster). We extracted qualitative data from quantitative studies on implementation limitations. We excluded studies, which only provided test accuracy statistics without evaluating the tests’ impact on patient-important outcomes and modelling studies. We also excluded editorials, opinion pieces, non-research reports, theoretical studies, secondary quantitative studies, reports, case studies, case series or abstracts with insufficient information or no full texts available, as the methodology of the studies could not be fully appraised.

#### Population and setting

We defined our population as people suspected of having malaria infection caused by any of the four human malaria parasites (*Plasmodium falciparum, P. malariae, P. ovale and P. vivax*) who reside in any sub-Saharan African country, regardless of age, sex or disease severity.

#### Intervention

We restricted studies for inclusion to those assessing mRDTs, regardless of the test type or the manufacturer.

#### Comparator

We included studies comparing mRDTs to microscopy, molecular diagnosis (PCR) or clinical/presumptive/routine diagnosis.

#### Outcomes

We included studies reporting on at least one or more patient-important outcomes. We adopted the conceptual framework for the classification of these outcomes as described by Schumacher *et al*.^16^ Further details regarding the classification are available in our protocol.^12^

- Measures of the diagnostic impact that indirectly assess the effect of mRDTs on the diagnostic process, such as time to diagnosis/turn-around time and prediagnostic loss to follow-up.
- Measures of the therapeutic impact that indirectly assess the effect of mRDTs on treatment decisions, such as time to treatment, pretreatment loss to follow-up, antimalarial/antibiotics prescription patterns and patient adherence to the test results.
- Measures of the health impact that directly assess the effect of mRDTs on the patient’s health, such as mortality, morbidity, symptom resolution, quality of life and patient health costs.

### Search methods for identifying studies

#### Electronic searches

Given the review’s purpose to assess the methodology of existing studies, we searched the following electronic databases for a representative sample till May 2022; MEDLINE, EMBASE, Cochrane Library and African Index Medicus. We also searched clinical trial registries, including clinicaltrials.gov, the meta-register of controlled trials, the WHO trials register and the Pan African Clinical Trials Registry. We applied a broad search strategy that included the following key terms: “Malaria”, “Diagnosis”, “Rapid diagnostic test”, “Impact”, “Outcome” and their associated synonyms. The full search strategy is provided in online supplemental file 2.

#### Other searches

We searched reference lists and citations of relevant systematic reviews that assessed the impact of mRDTs on patient-important outcomes. We checked for searches from conference proceedings within our search output.

### Study selection

Two reviewers independently screened the titles and abstracts of the search output and identified potentially eligible full texts using Covidence—an online platform for systematic reviews.^17^ We resolved any differences or conflicts through discussion among the reviewers or consulting a senior reviewer.

### Data extraction

Two reviewers independently extracted data from studies included using a predesigned and standard data extraction form in Covidence.^17^ We piloted the form on two potentially eligible studies before its use and resolved any differences or conflicts through a discussion among the reviewers or consulting a senior reviewer. The study information that was extracted included the following:

- General study details include the first author, year, title, geographical location(s), population, target condition and disease seasonality.
- Study design details such as the type of study, intervention, comparator, prediagnostic, pretreatment and post-treatment loss to follow-up, outcome measures and results for outcome measures (effect size and precision). Study design issues were also considered, including sample size, study setting, inclusion criteria and study recruitment.
- The quality assessment of the included studies was also performed using the National Institute for Health (NIH) quality assessment tools^18^ (online supplemental file 3).
- The implementation challenges, as reported by study authors in the methods and the discussion sections, were extracted according to the four main domains of the Supporting the Use of Research Evidence (SURE) framework for identifying barriers and enablers to health systems: recipient of care, providers of care, health system constraints and sociopolitical constraints^19^ (online supplemental file 4).

### Quality assessment

We assessed the methodological quality of included studies in Covidence.^17^ We adopted two NIH quality assessment tools^18^ for experimental and observational designs. Two reviewers independently assessed the methodological quality of studies as stratified by study design. We resolved any differences or conflicts by discussing among the reviewers or consulting a senior reviewer. Our quality evaluation was based on the number of quality criteria a study reported about its internal validity. The overall score was used to gauge the study’s methodological quality. We did not exclude studies based on the evaluation of methodological quality. Instead, we used our assessment to explain the methodological issues affecting impact studies of mRDTs.

### Analysis

We did not pool results from included individual studies, but we conducted descriptive statistics by synthesising our results narratively and graphically, as this was a methodological review. All included studies were thereby considered during narrative synthesis.

#### Quantitative data

We started our analysis by listing and classifying identified study designs and patient-important outcomes according to similarities. Stratified by study design, we used descriptive statistics for summarising key study characteristics. Descriptive analysis was done using STATA V.17 (Stata Corp, College Station, TX).

#### Qualitative data

We used the thematic framework analysis approach to analyse and synthesise the qualitative data to enhance our understanding of why the health stakeholders thought, felt and behaved as they did.^20^ We applied the following steps: familiarisation with data, selection of a thematic framework (SURE),^19^ coding themes, charting, mapping and interpreting identified themes.

## Results

### Study selection

**Figure 1:**
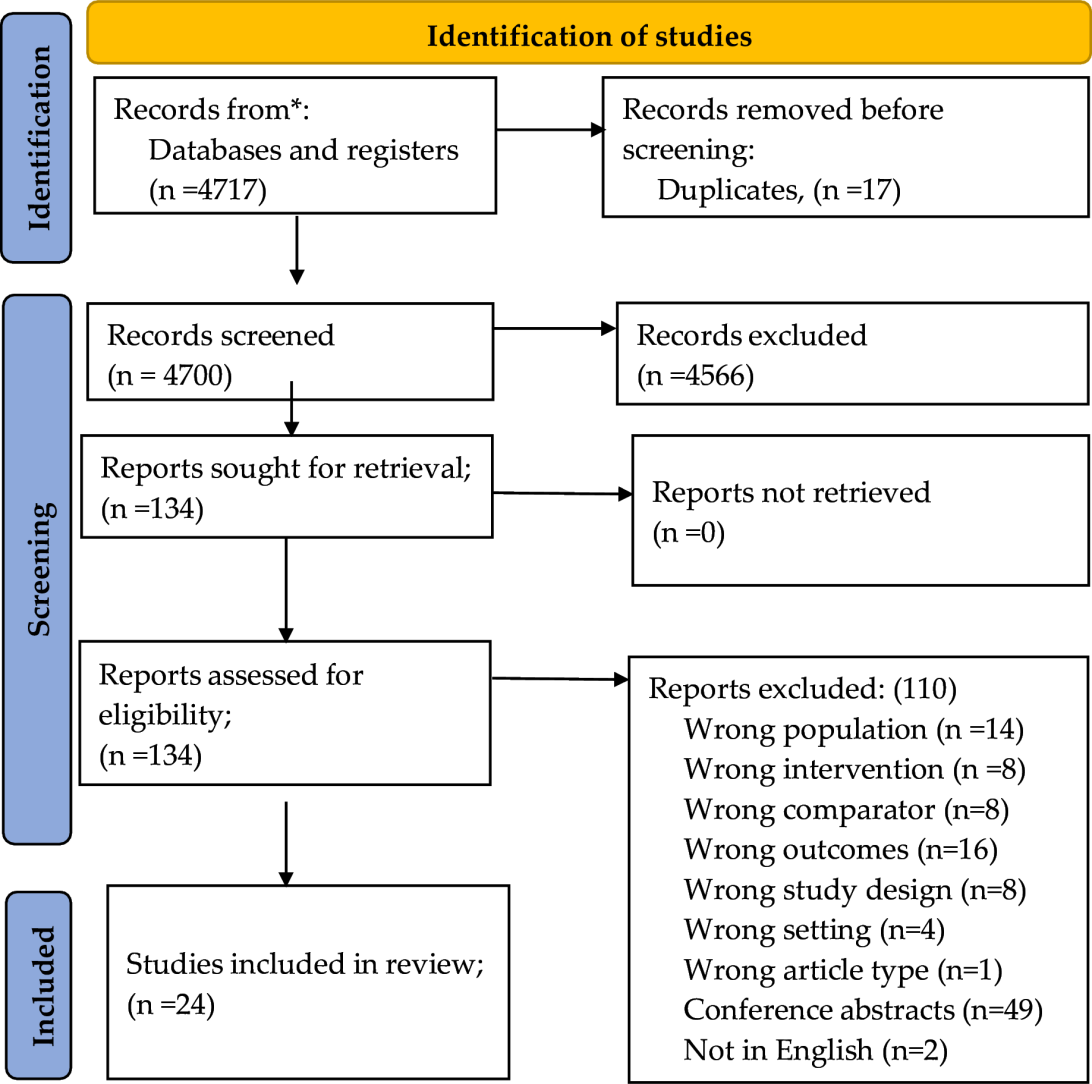
Preferred Reporting Items for Systematic Reviews and Meta-Analyses 2020 flow diagram showing the study selection process.

A summary of our study selection has been provided in figure 1. Our search yielded 4717 records as of June 2022. After removing 17 duplicates, we screened 4700 studies based on their titles and abstracts and excluded 4566 records. After that, we retrieved 134 full texts and screened them against the eligibility criteria. We excluded 110 studies. The characteristics of excluded studies are shown in online supplemental file 5. Therefore, we included 24 studies in this systematic review.

### General characteristics of included studies

Study characteristics have been summarised in online supplemental file 6. Studies included in this review were done in Ghana (7, 29.2%), Uganda (7, 29.2%), Tanzania (6, 25%), Burkina Faso (3, 12.5%), Nigeria (2, 8.3%) and Zambia (1, 4.2%). Most studies (16, 66.7%) were done on mixed populations of children and adults, while the remaining (8, 33.3%) were done on children alone. All studies (24, 100%) tested mRDTs as the intervention. Most studies (18, 75%) compared mRDTs to presumptive treatment/clinical diagnosis/clinical judgement, while the remaining (7, 29.2%) had microscopy and routine care (1, 4.2%) as their comparator. No study reported on PCR as a control.

Of all included studies, (17, 70.8%) were carried out in rural areas within government-owned facilities, (7, 29.2%) in urban areas and (2, 8.3%) in peri-urban areas. Few studies (6, 25%) were conducted in privately owned propriety facilities. Most studies (15, 62.5%) were conducted in health facilities and only (9, 37.5%) were within the communities. Studies conducted within health centres were (9, 37.5%), while those conducted in hospitals were (7, 29.2%). Most studies (15, 62.5%) were conducted during the high malaria transmission season, (9, 37.5%) during the low malaria season and (4, 16.7%) during the moderate malaria season. *P. falciparum* was the most common malaria parasite species (21, 87.5%)

### Study designs

We included multiple-armed studies with an intervention and a comparator (online supplemental file 6). Of the 24 studies, (15, 62.5%) were experimental designs in which, (10, 41.7%) were cluster randomised controlled trials (4, 16.7%) were individual randomised controlled trials and (1, 4.2%) was a randomised crossover trial. Of the remaining studies, (5, 20.8%) were quasi-experimental designs (non-randomised studies of intervention) in which (4, 16.7%) were pre-post/before and after studies and (1, 4.2%) was non-randomised crossover trials. The remaining studies (4, 16.7%) were observational where, (3, 12.5%) were cross-sectional designs and (1, 4.2%) was a cohort study.

### Patient-important outcomes

Patient-important outcome measures and individual study findings are summarised in online supplemental file 7. Of the 24 included studies, (21, 87.5%) measured the therapeutic impact of mRDTs, while (13, 54.2%) evaluated its health impact and only (1, 4.2%) assessed its diagnostic impact. Only (13, 54.2%) of all studies reported statistically significant findings.

#### Measures of therapeutic impact

Of the included studies, (20, 83.3%) reported on either antimalarials or antibiotics prescription patterns. The patient’s adherence to test results was reported by (3, 12.5%) studies, and the time taken to initiate treatment was reported by (2, 8.3%). In contrast, the pretreatment loss to follow-up was reported by (1, 4.2%) study. Studies reporting statistically significant findings on prescription patterns were (12, 50%), in which (11, 45.8%) demonstrated mRDTs’ potential to reduce over-prescription of antimalarials. In contrast, (1, 4.2%) study reported increased antimalarial prescription in the mRDT arm. Other statistically significant findings were reported by two studies where (1, 4.2%) reported that patients’ adherence to test results was poor in the malaria RDT arm. In contrast, the other (1, 4.2%) reported that mRDTs reduced the time to offer treatment.

#### Measures of health impact

Of the included studies, (6, 25%) reported on mortality, while (5, 20.8%) reported on symptom resolution. Patient health cost was reported by (4, 16.7%) studies, while patient referral and clinical re-attendance rates were reported by (2, 8.3%) each. Few (3, 12.5%) studies reported statistically significant findings on measuring the health impact that mRDTs improved the patient’s health outcomes by reducing morbidity.

#### Measures of diagnostic impact

Time taken to diagnose patients with malaria was reported by (1, 4.2%) study where diagnosis using mRDTs reduced the time to diagnose patients, but the findings were not statistically significant.

### Implementation challenges

The themes identified among included studies according to the SURE framework^19^ are presented in table 1. Most themes (n=7, 50%) emerged from the health system constraints domain while only one theme was reported under the domain, social and political constraints. Two themes, human resources and patient’s attitude were dominant. Lack of qualified staff in some study sites and patient’s preference for alternative diagnostic tests other than mRDTs hindered effective implementation of five studies.

**Table 1:** Implementation challenge reported by the included studies

No	Theme	Explanation
	**Domain 1: health system constraints**	
1	Human resources	Three studies^26^,^28^ reported on this theme. There were few qualified medical staff who were consequently overworked
2	Facilities	Two studies reported this theme,^29 30^ where the lack of essential facilities and diagnostic resources was predominant, especially in rural settings
3	Financial resources	One study^31^ reported receiving little funding to support the project
4	Clinical supervision	One study^32^ reported poor supervision of the healthcare workers, resulting in study protocol deviations and, ultimately, over-prescription practices
5	Education system	One study^33^ reported on varying education levels of professionals, especially at the lower levels of care, that could have contributed to poor treatment decisions offered to the patients based on intuition rather than test results
6	Management and leadership	One study^31^ highlighted the poor coordination between different health systems within multiple study sites, given the multicentre setting
7	Procurement and distribution systems	One study^34^ reported mRDTs and ACT stock-outs resulting from procurement concerns
	**Domain 2: recipients of care (patients)**	
8	Attitude	Three studies reported this theme.^27 33 35^ Issues regarding patients’ unacceptability of mRDTs resulted in preferences for alternative diagnostic tests to mRDTs
9	Knowledge and skills	Two studies reported challenges under this theme.^27 33^ There was little consumer knowledge of the mRDTs
10.	Motivation	One study^36^ reported that most of their patients practiced self-treatment due to self-drive to accept the results of the mRDTs, especially when negative and no antimalarials were issued
	**Domain 3: providers of care (healthcare practitioners)**	
11.	Attitude	Two studies^32 33^ reported on this theme. Concerns about providers’ low confidence in the negative mRDTs’ readings because of the poor perceptions regarding the credibility of mRDTs came out strongly
12.	Knowledge and skills	Two studies^27 32^ reported challenges under this theme. The expertise required to conduct the malaria tests was inadequate in resource-limited settings
13.	Motivation	One study^37^ reported that most healthcare workers showed partial acceptance and adoption of mRDTs in preference to microscopy
	**Domain 4: social and political constraints**	
14.	Short-term thinking	One study^31^ conducted within the community pharmacy setting showed that some participating private drug retailers had short-term momentary perceptions about the cost-effectiveness of mRDTs and sold-out mRDTs in both study arms (intervention and comparator)

ACTsartemisinin-based combination therapiesmRDTsmalaria rapid diagnostic tests

### Methodological quality of included studies

**Figure 2:**
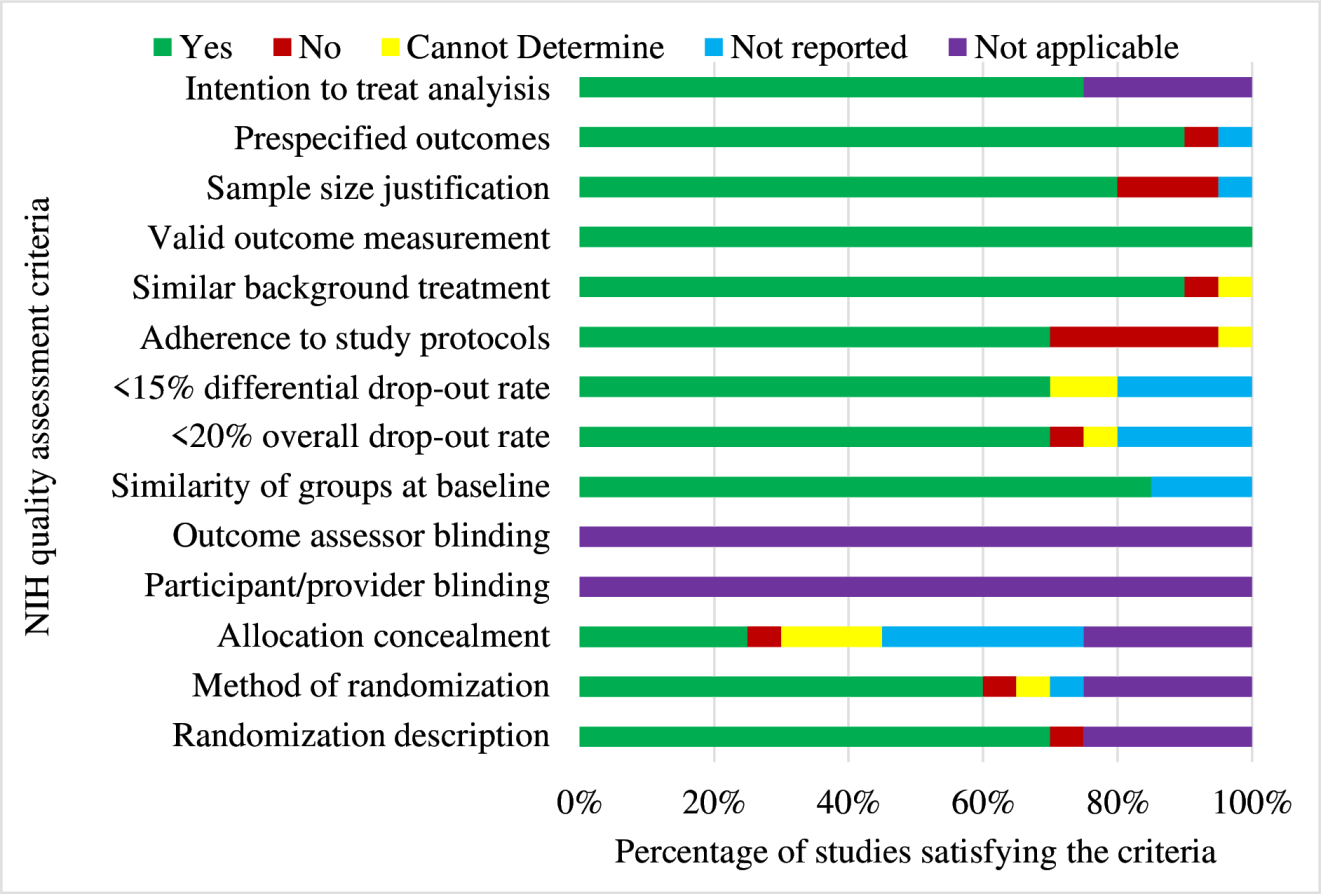
Quality assessment of controlled intervention study designs. NIH, National Institute for Health.

The methodological quality of the included studies is summarised in figures2  3. All studies assessed their outcomes validly and reliably and consistently implemented them across all participants. Some studies did not provide adequate information about loss-to-follow-up. Overall, (17, 70.8%) were of good methodological quality in which (11, 45.8%) were experimental, (3, 12.5%) were quasi-experimental and (3, 12.5%) were observational studies; however, blinding was not feasible. Concerns regarding patient non-adherence to treatment were reported in some studies. Sample size justification which is crucial when detecting differences in the measured primary outcomes was poorly reported among most studies. A detailed summary of each study’s performance is available in online supplemental files 8 and 9.

**Figure 3:**
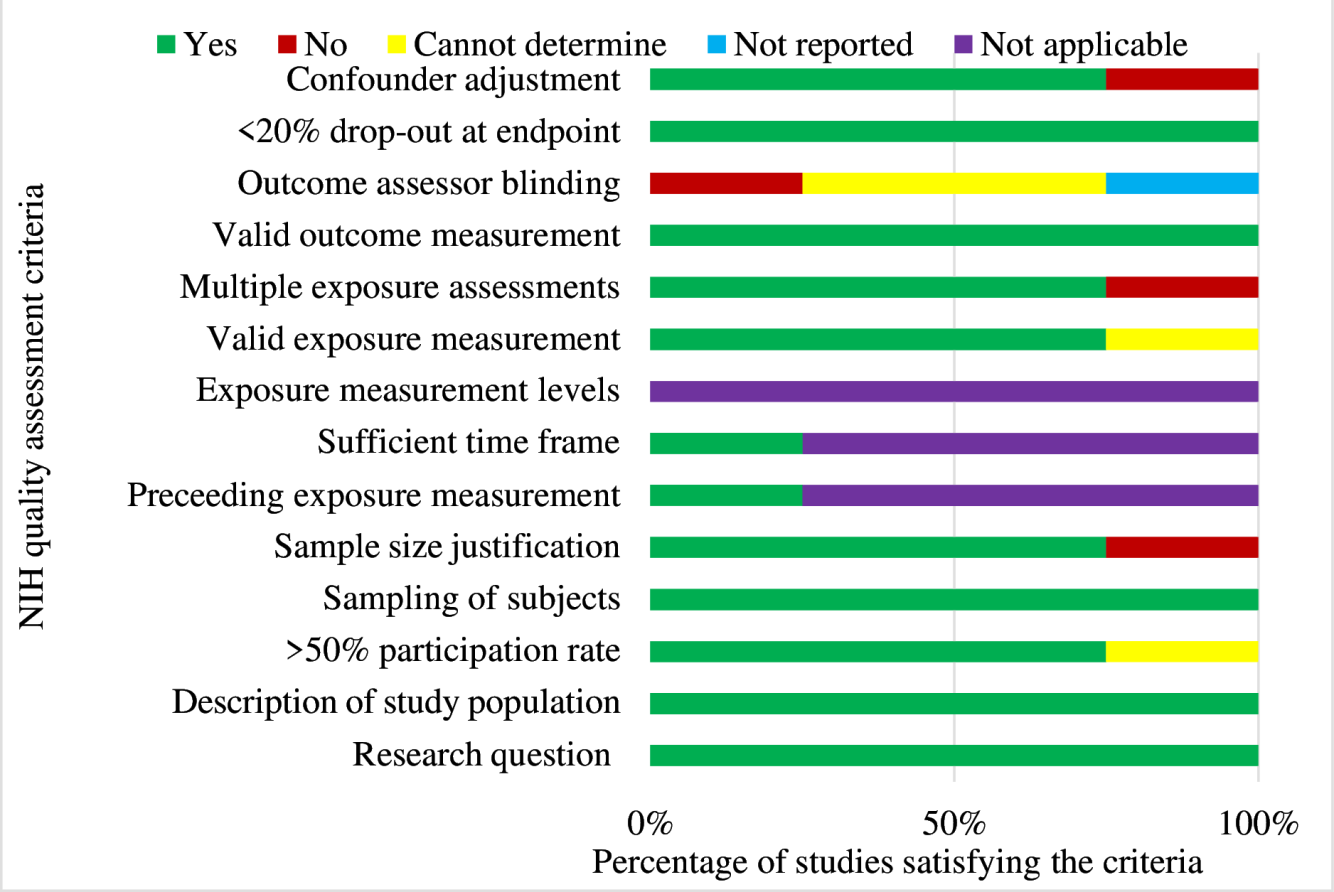
Quality assessment of observational study designs. NIH, National Institute for Health.

## Discussion

In this methodological systematic review, we assessed the designs, patient-important outcomes, implementation challenges and the methodological quality of studies evaluating the impact of mRDTs on patient-important outcomes within sub-Saharan Africa. We found evidence of mRDTs’ impact on patient-important outcomes came from just a few (six) from Western, Eastern and Southern African countries. Few studies were done on children, while most enrolled mixed populations in rural settings within government-owned hospitals. Few studies were conducted within the community health posts. Included studies assessed mRDTs’ impact compared with either microscopy/clinical diagnosis, with a majority being carried out during the high malaria transmission seasons in areas predominated by *P. falciparum*. Studies included were primary comparative designs, with experimental designs being the majority, followed by quasi-experimental and observational designs.

While most studies evaluated the therapeutic impact of mRDTs by measuring the prescription patterns of antimalarials/antibiotics, few assessed the test’s health and diagnostic impact. Few studies reported statistically significant findings, mainly on reduced antimalarial prescription patterns due to mRDTs. Most studies were of good quality, but quality concerns were lack of adequate information about loss-to-follow-up, inability to blind participants/providers/investigators, patient’s poor adherence to treatment options provided as guided by the predefined study protocols and lack of proper sample size justification. Key implementation limitations included inadequate human resources, lack of facilities, patients’ unacceptability of mRDTs, little consumer knowledge of the test and the providers’ low confidence in mRDTs’ negative results.

Schumacher *et al* conducted a similar study focusing on the impact of tuberculosis molecular tests, but unlike ours, they did not focus on implementation challenges. Similar to our results, Schumacher *et al*^16^ identified that evidence of the impact of diagnostic tests comes from just a small number of countries within a particular setting.^16^ Likewise, most studies evaluating the impact of diagnostic tests are done in health facilities like hospitals rather than in the community.^16^ Our finding that the choice of study design in diagnostic research is coupled with trade-offs is in line with Schumacher’s review.^16^ In the same way, experimental designs are mostly preferred in assessing diagnostic test impact, followed by quasi-experimental studies—majorly pre-post studies—conducted before and after the introduction of the intervention.^16^ Our findings also agree that observational designs are the least adopted in evaluating diagnostic impact.^16^ Similarly, our review’s finding concur with Schumacher *et al* that it may be worthwhile to explore other designs^16^ that use qualitative and quantitative methods, that is, the mixed-methods design, as this can create a better understanding of the test’s impact in a pragmatic way.

Our findings that studies indirectly assess the impact of diagnostic tests on patients by measuring the therapeutic impact rather than the direct health impact agree with Schumacher *et al*.^16^ However, in this systematic review, the ‘prescription patterns’ were most reported in contrast to Schumacher *et al*, where the ‘time to treatment’ was by far the most common.^16^ Similar to our finding, Schumacher *et al* determined that there is a trade-off in the choice of design and the fulfilment of criteria set forth to protect the study’s internal validity.^16^ While Schumacher *et al* investigated the risk of bias, our review focused on methodological quality.^16^

Diagnostic impact studies are complex to implement despite being crucial to any health system seeking to roll-out the universal health coverage programmes.^21^ Unlike therapeutic interventions that directly affect outcomes, several factors influence access to and effective implementation of diagnostic testing.^22^ While it is easier to measure indirect upstream outcomes to quantify mRDTs’ impact on diagnosis and treatment options, it is crucial to understand the downstream measures such as morbidity (symptom resolution, clinical re-attendance and referrals), mortality, patient health costs^22^ are key to improving value-based care. Contextual factors such as the provider’s lack of trust in the test’s credibility can negate the positive effects of the test, such as good performance. This is a problem facing health systems that are putting up initiatives to roll out mRDTs as the providers often perceive that negative mRDTs’ results are false positives.^16 22^ Consequently, lacking essential facilities and human resources can hinder the true estimation of the value mRDTs contribute to the patient’s health in resource-limited areas.

### Strengths and limitations

We conducted a robust literature search to get a recent representative sample of articles to assess the methodology. In addition to the methodology of studies, we evaluated the implementation challenges that limit the effect of the tests. Although we only included studies published in English which could affect generalisability of these findings, we believe this is a representative sample. Included studies were just from a few countries with sub-Sahara which could limit generalisability to other countries within the region. Since the overall sample size may not be an adequate representative of the entire population, the findings presented herein should be interpreted with caution. Additionally, considerations of the limited diversity in terms of study populations, interventions and outcome measures due to the few countries represented in the review should be included when interpreting our findings.

Health system concerns in both anglophone and francophone countries in sub-Saharan Africa are similar.^23^ Studies did not report on blinding, but this did not affect their methodological quality since prior knowledge of the test and the intervention itself calls for having prior knowledge of the test. Our study was limited by reporting of study items such as randomisation and blinding of participants, providers and outcome assessors. This limited our quality assessment in quasi-experimental studies. Therefore, authors are encouraged to report the study findings according to the relevant reporting guidelines.^24^ Most studies did not justify their sample sizes which could have compromised the validity of findings by influencing the precision and reliability of estimates. In cases where the sample size is inadequate, the reliability and generalisability of the findings becomes limited due to imprecise estimates with broad CIs. Studies reported poor adherence to protocols which could have reduced the sample size and the overall statistical power which could limit validity.

### Implications for practice, policy and future research

Controlling the malaria epidemic in high-burden settings in sub-Saharan Africa will require the effective implementation of tests that do more than provide incremental benefit over current testing strategies. Contextual factors affecting the test performance need to be considered a priori and factors introduced to mitigate their effect on implementing mRDTs. Process evaluations^25^ can be incorporated into quantitative studies or done alongside quantitative studies to determine whether the tests have been implemented as intended and resulted in certain outputs. Process evaluations^25^ can be incorporated into experimental studies to assess contextual challenges that could influence the design. Process evaluations can help decision-makers ascertain whether the mRDTs could similarly impact the people if adopted in a different context. Therefore, not only should process evaluations be performed but they should also be performed in a variety of contexts. It is prudent that patient-important outcomes be measured alongside process evaluations to better understand how to implement mRDTs. It may be worthwhile to focus on methodological research that guides impact evaluation reporting, particularly those that consider contextual factors. Future studies on the impact of mRDTs could improve by conducting mixed-methods designs which might provide richer data interpretation and insights into implementation challenges. Future studies could also consider providing clear justification for the sample size to ensure there is enough power to detect a significant difference.

## Conclusion

Most studies evaluating mRDTs’ impact on patient-important outcomes in sub-Saharan Africa are randomised trials of good methodological quality conducted in real-life settings. The therapeutic effect of mRDTs is by far the most common measure of mRDTs’ impact. Quality issues include poor reporting on sample size justification and reporting of statistically significant findings. Effective studies of patient-important outcome measures need to account for contextual factors such as inadequate resources, patients’ unacceptability of mRDTs, and the providers’ low confidence in mRDTs’ negative results, which hinder the effective implementation of impact-evaluating studies. Process evaluations can be incorporated into experimental studies to assess contextual challenges that could influence the design.

## supplementary material

10.1136/bmjopen-2023-077361 online supplemental file 1

10.1136/bmjopen-2023-077361 online supplemental file 2

## Data Availability

Data are available upon reasonable request.
