# Supplementary material for: Evaluating the impact of malaria rapid diagnostic tests on patient-important outcomes in sub-Saharan Africa: a systematic review of study methods to guide effective implementation
Source: BMJ Open. 2024 Sep 10;14(9):e077361. doi: 10.1136/bmjopen-2023-077361 (PMC11409401; doi:10.1136/bmjopen-2023-077361)
Supplement: online supplemental file 1 [file bmjopen-14-9-s001.pdf]

Supplementary file 2: Full search strategies

Part 1: MEDLINE (PubMed) Search Strategy

Search set	MEDLINE (PubMed) <from inception up to 02 May 2022>
**1**	Exp Malaria[MeSH]
**2**	Exp Plasmodium [MeSH]
**3**	Malaria [Title/Abstract]
**4**	1 or 2 or 3
**5**	Exp Reagent kits, diagnostics [MeSH]
**6**	rapid diagnos* test* [Title/Abstract]
**7**	RDT* [Title/Abstract]
**8**	"point of care" [Title/Abstract]
**9**	Dipstick* [Title/Abstract]
**10**	Rapid diagnos* device* [Title/Abstract]
**11**	MRDT [Title/Abstract]
**12**	OptiMal [Title/Abstract]
**13**	Binax NOW [Title/Abstract]
**14**	ParaSight [Title/Abstract]
**15**	Rapid test* [Title/Abstract]
**16**	Card test* [Title/Abstract]
**17**	Rapid AND (detection* or diagnos*) [Title/Abstract]
**18**	5 or 6 or 7 or 8 or 9 or 10 or 11 or 12 or 13 or 14 or 15 or 16 or 17
**19**	4 and 18
**20**	Mortality [Mesh] OR Morbidity [Mesh] OR Prognosis [Mesh]
**21**	Treatment Outcome [Mesh] OR Length of Stay [Mesh] or "Time-to-Treatment"[Mesh] or "Quality of Life"[Mesh]
**22**	"Cost-Benefit Analysis"[Mesh] or "cost-effectiveness "[Title/Abstract]
**23**	endpoint* OR outcome* OR mortality OR prognosis OR prognostic or burden or "case detection" or "time to diagnosis" [Title/Abstract]
**24**	impact* OR effect* or "treatment initiation" OR benefit* or "birth weight" or "adverse events" or safety [Title/Abstract]
**25**	Prescription* or prescribing or fever or "case management" or anti-malarial* or antimalarial* or antibiotic* or compliance or Follow-up or "empirical treatment" or "syndromic treatment" [Title/Abstract]
**26**	Antimalarials/administration & dosage/therapeutic use [Mesh]
**27**	Perception* or experience* or feasibility or acceptance or acceptability [Title/Abstract]
**28**	Drug Prescriptions [Mesh] or "Medication Adherence"[Mesh] or "Patient Acceptance of Health Care"[Mesh]
**29**	20 or 21 or 22 or 23 or 24 or 25 or 26 or 27 or 28
**30**	18 and 29

Part 2: EMBASE Search Strategy

Search Set	EMBASE <1996 to 2022 week 17>
**1**	malaria/ or malaria.mp.
**2**	Plasmodium/ or plasmodium.mp.
**3**	1 or 2
**4**	("rapid diagnos* test*" or RDT* or dipstick).ab. or ("rapid diagnos* test*" or RDT* or dipstick).ti.

**5**	("point of care" or "Rapid diagnos* device* " or MRDD or "Binax NOW " or ParaSight or "Rapid test* " or "card test*").ab.
**6**	("point of care" or "Rapid diagnos* device* " or MRDD or "Binax NOW " or ParaSight or "Rapid test* " or "card test*").ti.
**7**	("molecular diagnosis" or "molecular diagnostics").ti. or ("molecular diagnosis" or "molecular diagnostics").ab.
**8**	4 or 5 or 6 or 7
**9**	3 and 8
**10**	(mortality or morbidity or prognosis).ti. or (mortality or morbidity or prognosis).ab.
**11**	outcome*.ti. or outcome*.ab.
**12**	quality of life.mp. or "quality of life"/
**13**	"Costs and Cost Analysis"/
**14**	(endpoint* or burden or "case detection" or "time to diagnosis").ti. or (endpoint* or burden or "case detection" or "time to diagnosis").ab.
**15**	(impact* or effect* or "treatment initiation" or benefit* or " birth weight" or " adverse events" or safety).ti. or (impact* or effect* or "treatment initiation" or benefit* or " birth weight" or " adverse events" or safety).ab.
**16**	(Prescription* or prescribing or fever or " case management" or anti-malarial* or antimalarial* or antibiotic* or compliance or Follow-up or " empirical treatment" or "syndromic treatment").ti. or (Prescription* or prescribing or fever or " case management" or anti-malarial* or antimalarial* or antibiotic* or compliance or Follow-up or " empirical treatment" or "syndromic treatment").ab.
**17**	(Perception* or experience* or feasibility or acceptance or acceptability).ti. or (Perception* or experience* or feasibility or acceptance or acceptability).ab.
**18**	10 or 11 or 12 or 13 or 14 or 15 or 16 or 17
**19**	9 and 18

Part 3: Cochrane Library Search Strategy

Search set	Cochrane Library <issue 4 of 12, April 2022>
**1**	malaria:ti,ab,kw or plasmodium:ti,ab,kw
**2**	"rapid diagnos* test*" or RDT* or dipstick:ti,ab,kw
**3**	"point of care" or "Rapid diagnos* device* " or MRDD:ti,ab,kw or "Binax NOW " or ParaSight or "Rapid test* " or "card test*":ti,ab,kw or Rapid and (detection* or diagnos*):ti,ab,kw
**4**	MeSH descriptor: [Reagent Kits, Diagnostic] explode all trees
**5**	#2 or #3 or #4
**6**	#1 and #5
**7**	MeSH descriptor: [Mortality] explode all trees
**8**	MeSH descriptor: [Morbidity] explode all trees
**9**	MeSH descriptor: [Prognosis] explode all trees
**10**	MeSH descriptor: [Treatment Outcome] explode all trees
**11**	MeSH descriptor: [Length of Stay] explode all trees
**12**	MeSH descriptor: [Time-to-Treatment] explode all trees
**13**	MeSH descriptor: [Quality of Life] explode all trees

**14**	MeSH descriptor: [Cost-Benefit Analysis] explode all trees
**15**	cost-effectiveness:ti,ab,kw (Word variations have been searched)
**16**	endpoint* or outcome* or mortality or prognosis or prognostic or burden or "case detection" or "time to diagnosis":ti,ab,kw
**17**	impact* or effect* or "treatment initiation" or benefit* or "birth weight" or "adverse events" or safety:ti,ab,kw
**18**	Prescription* or prescribing or fever or "case management" or anti-malarial* or antimalarial* or antibiotic* or compliance or Follow-up or "empirical treatment" or "syndromic treatment":ti,ab,kw
**19**	MeSH descriptor: [Antimalarials] explode all trees
**20**	Perception* or experience* or feasibility or acceptance or acceptability:ti,ab,kw (Word variations have been searched)
**21**	MeSH descriptor: [Drug Prescriptions] explode all trees
**22**	MeSH descriptor: [Medication Adherence] explode all trees
**23**	MeSH descriptor: [Patient Acceptance of Health Care] explode all trees
**24**	#7 or #8 or #9 or #10 or #11 or #12 or #13 or #14 or #15 or #16 or #17 or #18 or #19 or #20 or #21 or #22 or #23
**25**	#6 and #24

Part 4: Africa Index Medicus Search strategy

Malaria or plasmodium [Words] and diagnosis or diagnostic or RDT$ [Words] and endpoint$ or outcome$ or mortality or prognosis or prescription$ or attitude$ or experience or perception or benefit [Words]

Part 5: Clinical Trial Registries Search Strategies

- Clinicaltrials.gov

  Rapid diagnostic test | Malaria

- WHO ICTRP

  Malaria and (rapid diagnostic test* or RDT*)

- Meta-register of controlled trials (mRCT)

  Malaria and (rapid diagnostic test* or RDT*)

- Pan African Clinical Trials Registry

  Malaria and (rapid diagnostic test* or RDT*)

Supplementary file 3: National Institute of Health (NIH) tool used to assess the methodological quality of included studies

Part 1: Quality assessment of controlled intervention studies

Criteria	Description
Was the study described as randomized, a randomized trial, a randomized clinical trial, or an RCT?	Was the study described as randomized? A study does not satisfy quality criteria as randomized simply because the authors call it randomized; however, it is a first step in determining if a study is randomized
Was the method of randomization adequate (i.e., use of randomly generated assignment)?	Adequate randomization: Randomization is adequate if it occurred according to the play of chance (e.g., computer generated sequence in more recent studies, or random number table in older studies). If assignment is not by the play of chance, then the answer to this question is no.
Was the treatment allocation concealed (so that assignments could not be predicted)?	This means that one does not know in advance, or cannot guess accurately, to what group the next person eligible for randomization will be assigned. Methods include sequentially numbered opaque sealed envelopes, numbered or coded containers, central randomization by a coordinating centre, computer-generated randomization that is not revealed ahead of time, etc.
Were study participants and providers blinded to treatment group assignment?	Blinding means that one does not know to which group–intervention or control–the participant is assigned. It is also sometimes called "masking." The reviewer assessed whether each of the following was blinded to knowledge of treatment assignment: (1) the person assessing the primary outcome(s) for the study; (2) the person receiving the intervention; and (3) the person providing the intervention.<br>Sometimes the individual providing the intervention is the same person performing the outcome assessment. This should be noted.
Were the people assessing the outcomes blinded to the participants' group assignments?	
Were the groups similar at baseline on important characteristics that could	This question relates to whether the intervention and control groups have similar baseline characteristics on average especially those characteristics that may affect the

affect outcomes (e.g., demographics, risk factors, co-morbid conditions)?	intervention or outcomes. The point of randomized trials is to create groups that are as similar as possible except for the intervention(s) being studied in order to compare the effects of the interventions between groups. When reviewers abstracted baseline characteristics, they noted when there was a significant difference between groups.
Was the overall drop-out rate from the study at endpoint 20% or lower of the number allocated to treatment?	"Dropouts" in a clinical trial are individuals for whom there are no end point measurements, often because they dropped out of the study and were lost to follow up.<br>Generally, an acceptable overall dropout rate is considered 20 percent or less of participants who were randomized or allocated into each group. An acceptable differential dropout rate is an absolute difference between groups of 15 percentage points at most (calculated by subtracting the dropout rate of one group minus the dropout rate of the other group).
Was the differential drop-out rate (between treatment groups) at endpoint 15 percentage points or lower?	
Was there high adherence to the intervention protocols for each treatment group?	Did participants in each treatment group adhere to the protocols for assigned interventions? For example, if one group that was assigned to receive a particular drug at a particular dose had a large percentage of participants who did not end up taking the drug or the dose as designed in the protocol.
Were other interventions avoided or similar in the groups (e.g., similar background treatments)?	Changes that occur in the study outcomes being assessed should be attributable to the interventions being compared in the study. If study participants receive interventions that are not part of the study protocol and could affect the outcomes being assessed, and they receive these interventions differentially, then there is cause for concern because these interventions could bias results.
Were outcomes assessed using valid and reliable measures, implemented	What tools or methods were used to measure the outcomes in the study? Were the tools and methods accurate and reliable–for example, have they been validated, or are they objective? This is important as it indicates the confidence you can have

consistently across all study participants?	in the reported outcomes. Perhaps even more important is ascertaining that outcomes were assessed in the same manner within and between groups.
Did the authors report that the sample size was sufficiently large to be able to detect a difference in the main outcome between groups with at least 80% power?	Generally, a study's methods section will address the sample size needed to detect differences in primary outcomes. The current standard is at least 80 percent power to detect a clinically relevant difference in an outcome using a two-sided alpha of 0.05.
Were outcomes reported or subgroups analysed prespecified (i.e., identified before analyses were conducted)?	Investigators should pre specify outcomes reported in a study for hypothesis testing–which is the reason for conducting an RCT. Without prespecified outcomes, the study may be reporting ad hoc analyses, simply looking for differences supporting desired findings. Investigators also should pre specify subgroups being examined.
Were all randomized participants analysed in the group to which they were originally assigned, i.e., did they use an intention-to-treat analysis?	Intention-to-treat (ITT) means everybody who was randomized is analysed according to the original group to which they are assigned. This is an extremely important concept because conducting an ITT analysis preserves the whole reason for doing a randomized trial; that is, to compare groups that differ only in the intervention being tested.

Part 2: Quality assessment tool for observational cohort and cross-sectional studies

Criteria	Description
Was the research question or objective in this paper clearly stated?	Did the authors describe their goal in conducting this research? Is it easy to understand what they were looking to find? This issue is important for any scientific paper of any type. Higher quality scientific research explicitly defines a research question.
Was the study population clearly specified and defined?	Did the authors describe the group of people from which the study participants were selected or recruited, using demographics, location, and time period? If fewer than 50%

Was the participation rate of eligible persons at least 50%?	of eligible persons participated in the study, then there is concern that the study population does not adequately represent the target population. This increases the risk of bias.
Were all the subjects selected or recruited from the same or similar populations (including the same time period)? Were inclusion and exclusion criteria for being in the study prespecified and applied uniformly to all participants?	Were the inclusion and exclusion criteria developed prior to recruitment or selection of the study population? Were the same underlying criteria used for all of the subjects involved? This issue is related to the description of the study population, above, and you may find the information for both of these questions in the same section of the paper. If the study recruits groups from different clinic populations, then it will be a "no."
Was a sample size justification, power description, or variance and effect estimates provided?	Did the authors present their reasons for selecting or recruiting the number of people included or analysed? Do they note or discuss the statistical power of the study? This question is about whether or not the study had enough participants to detect an association if one truly existed.
For the analyses in this paper, were the exposure(s) of interest measured prior to the outcome(s) being measured?	This question is important because, in order to determine whether an exposure causes an outcome, the exposure must come before the outcome. For some prospective cohort studies, the investigator enrols the cohort and then determines the exposure status of various members of the cohort. Cross-sectional studies are conducted, where the exposures and outcomes are measured during the same timeframe hence, the answer to Question 6 should be "no."
Was the timeframe sufficient so that one could reasonably expect to see an association between exposure and outcome if it existed?	Did the study allow enough time for a sufficient number of outcomes to occur or be observed, or enough time for an exposure to have a biological effect on an outcome? Cross-sectional analyses allow no time to see an effect, since the exposures and outcomes are assessed at the same time, so those would get a "no" response.

For exposures that can vary in amount or level, did the study examine different levels of the exposure as related to the outcome (e.g., categories of exposure, or exposure measured as continuous variable)?	If the exposure can be defined as a range (examples: drug dosage, amount of physical activity, amount of sodium consumed), were multiple categories of that exposure assessed? If there are only two possible exposures (yes/no), then this question should be given an "NA," and it should not count negatively towards the quality rating.
Were the exposure measures (independent variables) clearly defined, valid, reliable, and implemented consistently across all study participants?	Were the exposure measures defined in detail? Were the tools or methods used to measure exposure accurate and reliable. When exposures are measured with less accuracy or validity, it is harder to see an association between exposure and outcome even if one exists. Also as important is whether the exposures were assessed in the same manner within groups and between groups; if not, bias may result.
Was the exposure(s) assessed more than once over time?	Was the exposure for each person measured more than once during the course of the study period? Multiple measurements with the same result increase our confidence that the exposure status was correctly classified. Also, multiple measurements enable investigators to look at changes in exposure over time.
Were the outcome measures (dependent variables) clearly defined, valid, reliable, and implemented consistently across all study participants?	Were the outcomes defined in detail? Were the tools or methods for measuring outcomes accurate and reliable–for example, have they been validated or are they objective? This issue is important because it influences confidence in the validity of study results. Also important is whether the outcomes were assessed in the same manner within groups and between groups.
Were the outcome assessors blinded to the exposure status of participants?	Blinding means that outcome assessors did not know whether the participant was exposed or unexposed. It is also sometimes called "masking." Sometimes the person measuring the exposure is the same person conducting the outcome assessment. In this case, the outcome assessor

	would most likely not be blinded to exposure status because they also took measurements of exposures. If so, make a note of that in the comments section. Think about whether it is likely that the person(s) doing the outcome assessment would know (or be able to figure out) the exposure status of the study participants.
Was loss to follow-up after baseline 20% or less?	Higher overall follow-up rates are always better than lower follow-up rates, even though higher rates are expected in shorter studies, whereas lower overall follow-up rates are often seen in studies of longer duration. Usually, an acceptable overall follow-up rate is considered 80 percent or more of participants whose exposures were measured at baseline.
Were key potential confounding variables measured and adjusted statistically for their impact on the relationship between exposure(s) and outcome(s)?	Were key potential confounding variables measured and adjusted for, such as by statistical adjustment for baseline differences? Logistic regression or other regression methods are often used to account for the influence of variables not of interest. This is a key issue in cohort studies, because statistical analyses need to control for potential confounders, in contrast to an RCT, where the randomization process controls for potential confounders. All key factors that may be associated both with the exposure of interest and the outcome–that are not of interest to the research question–should be controlled for in the analyses

Supplementary file 4: Framework for Supporting the Use of Research Evidence (SURE) for identifying the implementation challenges facing studies that evaluate mRDTs' impact on patient-important outcomes

Level	Barriers and enablers	Description
**Recipients of care**	Knowledge and skills	Recipients of care may have varying degrees of knowledge about the healthcare issue or the intervention, or may not have the skills to apply this knowledge. E.g. People may be unaware that family planning services are available at their local clinic or may not have the skills to prepare oral rehydration therapy when its use has been recommended.
	Attitudes regarding programme acceptability, appropriateness and credibility	Recipients of care may have opinions about the healthcare issue and the intervention, including views about the acceptability and appropriateness of the intervention and the credibility of the provider and the healthcare system. E.g. People may not agree with the choice of intervention or may not trust the reasons behind it
	Motivation to change or adopt new behaviour	Recipients of care may have varying degrees of motivation to change behaviour or adopt new behaviours. E.g. they may be more or less motivated to seek care
**Providers of care**	Knowledge and skills	Providers may have varying degrees of knowledge about the healthcare issue or the intervention, or may not have the skills to apply this knowledge. E.g. health workers may be unaware of guidelines on tuberculosis treatment or may not have received training in the implementation of these guidelines
	Attitudes regarding programme acceptability,	Providers may have opinions about the healthcare issue and the intervention, including views about the acceptability and appropriateness of the intervention and the credibility of the provider and the

	appropriateness and credibility	healthcare system. E.g. health workers may not agree with the choice of intervention or may not trust the reasons behind it
	Motivation to change or adopt new behaviour	Providers may have varying degrees of motivation to change behaviour or adopt new behaviours. E.g., they may be more or less motivated to take on new tasks
**Other stakeholders (including other healthcare providers, community health committees, community leaders, programme managers, donors, policy makers and opinion leaders)**	Knowledge and skills	Other stakeholders may have varying degrees of knowledge about the healthcare issue or the intervention, or may not have the skills to apply this knowledge. E.g. a community leader may have insufficient knowledge of the benefits of exclusive breastfeeding or may not feel skilled in running community meetings to promote infant care
	Attitudes regarding programme acceptability, appropriateness and credibility	Other stakeholders' may have opinions about the healthcare issue or the intervention, including views about the acceptability and appropriateness of the intervention and the credibility of the provider and the healthcare system. E.g. stakeholders may not agree with the choice of intervention because of competing interests or priorities
	Motivation to change or adopt new behaviour	Other stakeholders may have varying degrees of motivation to change behaviour or adopt new behaviours. E.g. programme managers may not be motivated to deliver supervision to remote clinics
**Health system constraints**	Accessibility of care	The accessibility of healthcare facilities may affect implementation of the option, for instance because of financial (user fees), geographic (distance to clinic), or social (access for certain ethnic groups) factors
	Financial resources	Additional financial resources may be needed to implement the option

	Human resources	An increased supply or distribution of health workers may be needed to implement the option
	Educational system	The educational system for health workers may need to be modified
	Clinical supervision	Health workers may require more supervision than is currently provided to implement the option
	Internal communication	Changes in communication between different levels of the health system or between the health and social care systems may be needed to implement the option
	External communication	Changes in communication between health workers and recipients of care needs may be needed to implement the option
	Allocation of authority	Changes may be needed regarding the levels or individuals that have the authority to make decisions
	Accountability	Changes may be needed so that those with the authority to make decisions are accountable for the decisions they make
	Management and or leadership	Adequately trained managers or sufficient leadership may be needed to implement the option
	Information systems	Adequate information systems to assess and monitor needs, resource use, and utilisation of targeted services may be needed to implement the option
	Facilities	Adequate supply and distribution of necessary supplies and equipment to facilities, and maintenance of these facilities, may be needed to implement the option
	Patient flow processes	Adequate processes for outreach and receiving, referring and transferring

		patients may be needed to implement the option
	Procurement and distribution systems	Adequate systems for procuring and distributing drugs and other supplies may be needed to implement the option
	Incentives	Reimbursement systems for patients, health workers or others may need to be structured to facilitate rather than hinder implementation of the option
	Bureaucracy	Paperwork and procedures may need to be structured to facilitate rather than hinder implementation of the option
	Relationship with norms and standards	Current norms and standards of practice need to be in line with the relevant option
**Social and political constraints**	Ideology	Ideological beliefs (e.g. in 'free markets') may affect implementation of the option
	Short-term thinking	Implementation of the option may be opposed if its benefits are likely to occur beyond the time horizon of decision makers (e.g. after the next election)
	Contracts	Contracts with service providers or enforcement of contracts may not be adequate to ensure implementation of the option or the types of effective care at which it is targeted
	Legislation or regulations	Changes to legislation or regulations, including those that are general (e.g. regulating government contracts, regulating working conditions) and those that are specific to the health system (e.g. licensing health professionals) may be needed
	Donor policies	Donor policies and programmes may influence implementation
	Influential people	The opinions of influential people may influence the option or the types of effective care at which it is targeted

	Corruption	Corrupt behaviour by decision makers or others may influence implementation
	Political stability	Political instability may influence implementation

Supplementary file 5: Characteristics of Studies Excluded from our Review

Study ID	Reason for exclusion
Agaba 2015 (1)	Conference abstract
Agwu 2012 (2)	Conference abstract
Ansah 2011 (3)	Conference abstract
Audu 2016 (4)	Wrong intervention
Azikiwe 2012 (5)	Wrong patient population
Baiden 2012 (6)	Wrong study design
Baltzell 2019 (7)	Wrong comparator
Bisoffi 2011 (8)	Wrong study design
Boadu 2012 (9)	Conference abstract
Bottieau 2013 (10)	Wrong intervention
Boyce 2015 (11)	Wrong patient population
Boyce 2017 (12)	Wrong study design
Brasseur 2012 (13)	Conference abstract
Brigitte 2020 (14)	Conference abstract
Bruxvoort 2011 (15)	Conference abstract
Bruxvoort 2015 (16)	Conference abstract
Bruxvoort 2017 (17)	Wrong study design
Burchet 2017 (18)	Wrong study design
Catherine 2012 (19)	Conference abstract
Chanda 2009 (20)	Wrong outcomes
Chandler 2017 (21)	Wrong intervention
Chilongola 2015 (22)	Wrong intervention
Chinkhumba 2010 (23)	Wrong outcomes
Chukwu 2017 (24)	Conference abstract
Cohen 2015 (25)	Conference abstract
D'Acremont 2011 (26)	Wrong comparator
D'Acremont 2010 (27)	Wrong comparator
Das 2015 (28)	Conference abstract
De Carsalade 2009 (29)	Not in English
Eliades 2017 (30)	Conference abstract

Engo 2019 (31)	Conference abstract
Festo 2012 (32)	Conference abstract
Gerstl 2010 (33)	Wrong outcomes
Githinji 2018 (34)	Conference abstract
Gitonga 2012 (35)	Wrong outcomes
Gupta 2017 (36)	Wrong setting
Halliday 2014 (37)	Conference abstract
Halliday 2014 (38)	Wrong patient population
Hamer 2012 (39)	Wrong comparator
Hamer 2007 (40)	Wrong outcomes
Harchut 2013 (41)	Wrong outcomes
Harutyunyan 2010 (42)	Conference abstract
Herlihy 2016 (43)	Wrong article type
Hopkins 2017 (44)	Wrong study design
Houze 2009 (45)	Wrong patient population
Hsiang 2019 (46)	Wrong outcomes
Huth 2021 (47)	Wrong patient population
Ige 2014 (48)	Conference abstract
Ishengoma 2015 (49)	Conference abstract
Kalolella 2012 (50)	Conference abstract
Kamau 2020 (51)	Wrong intervention
Kapisi 2017 (52)	Conference abstract
Kariuki 2018 (53)	Conference abstract
Kiemde 2017 (54)	Conference abstract
Kipanga 2014 (55)	Wrong patient population
Kitutu 2017 (56)	Wrong intervention
Kochar 2010 (57)	Wrong outcomes
Kukula 2012 (58)	Conference abstract
Kumar 2011 (59)	Conference abstract
Lal 2016 (60)	Wrong outcomes
Lal 2015 (61)	Conference abstract
Lawrence 2014 (62)	Conference abstract

Leurent 2014 (63)	Conference abstract
Masanja 2012 (64)	Wrong patient population
Mawili-Mboumba 2010 (65)	Wrong study design
Matangila 2014 (66)	Conference abstract
Mbonye 2020 (67)	Wrong intervention
Mbonye 2014 (68)	Wrong intervention
Mbonye 2015 (69)	Conference abstract
Mbonye 2013 (70)	Conference abstract
Mbonye 2013 (71)	Conference abstract
Mfuh 2015 (72)	Conference abstract
Mosha 2010 (73)	Wrong comparator
Mubi 2013 (74)	Wrong patient population
Munier 2009 (75)	Not in English
Ndyomugyenyi 2012 (76)	Conference abstract
Ndyomugyenyi 2013 (77)	Conference abstract
Ndyomugyenyi 2013 (78)	Conference abstract
Ndyomugyenyi 2014 (79)	Conference abstract
Newman 2009 (80)	Conference abstract
Njau 2013 (81)	Conference abstract
Ojo 2020 (82)	Conference abstract
Okolo 2020 (83)	Conference abstract
Osei-Kwakye 2013 (84)	Wrong outcomes
Otshudiema 2013 (85)	Conference abstract
Oyeyemi 2015 (86)	Wrong outcomes
Parikh 2010 (87)	Wrong patient population
Pinto 1999 (88)	Wrong setting
Portugal 2017 (89)	Wrong patient population
Rantala 2010 (90)	Wrong outcomes
Reyburn 2004 (91)	Wrong patient population
Ruizendaal 2017 (92)	Conference abstract
Salomao 2015 (93)	Wrong comparator
Sangoro 2014 (94)	Wrong outcomes

Schrot-Sanyan 2013 (95)	Wrong patient population
Searle 2017 (96)	Conference abstract
Shakely 2013 (97)	Wrong comparator
Srinivasan 2000 (98)	Wrong setting
Swarthout 2007 (99)	Wrong outcomes
Tiruneh 2018 (100)	Wrong patient population
Uzochukwu 2009 (101)	Wrong patient population
VanEjik 2019 (102)	Conference abstract
Wang 2020 (103)	Conference abstract
Wernsdorfer 2009 (104)	Conference abstract
Williams 2008 (105)	Wrong outcomes
Wiseman 2014 (106)	Conference abstract
Wogu 2018 (107)	Wrong outcomes
Wongsrichanalai 2007 (108)	Conference abstract
Yimam 2022 (109)	Wrong study design
Zuninga 2015 (110)	Wrong setting

1. Agaba BB, Ojaku A, Streat E, Nuwa A, Okui P, Adibaku S, et al. Field-based quality monitoring of malaria rapid diagnostic tests in resource-limited settings: Experience from Uganda. American journal of tropical medicine and hygiene. 2015;93 (4 Supplement):73.
2. Agwu E, Kyarimpa M, Gulemye I. Abuse of antimalarial drugs in treatment of febrile patients with clinically diagnosed malaria in Bushenyi District, Uganda. Clinical Microbiology and Infection. 2012;18:55.
3. Ansah EK, Whitty CJ, Yeung S, Hansen K. Cost-effectiveness analysis of introducing rapid diagnostic tests (RDTs) for malaria diagnosis in public health centers where microscopy is available and peripheral clinics where only clinical diagnosis is available: The case of Ghana. American journal of tropical medicine and hygiene. 2011;1):350.
4. Audu R, Anto BP, Koffuor GA, Abruquah AA, Buabeng KO. Malaria rapid diagnostic test evaluation at private retail pharmacies in Kumasi, Ghana. J Res Pharm Pract. 2016;5(3):175-80.
5. Azikiwe CC, Ifezulike CC, Siminialayi IM, Amazu LU, Enye JC, Nwakwunite OE. A comparative laboratory diagnosis of malaria: microscopy versus rapid diagnostic test kits. Asian Pac J Trop Biomed. 2012;2(4):307-10.
6. Baiden F, Webster J, Tivura M, Delimini R, Berko Y, Amenga-Etego S, et al. Accuracy of rapid tests for malaria and treatment outcomes for malaria and non-malaria cases among under-five children in rural Ghana. PLoS One. 2012;7(4):e34073.
7. Baltzell K, Kortz TB, Scarr E, Blair A, Mguntha A, Bandawe G, et al. 'Not all fevers are malaria': a mixed methods study of non-malarial fever management in rural southern Malawi. Rural and remote health. 2019;19(2):4818.

8. Bisoffi Z, Sirima SB, Meheus F, Lodesani C, Gobbi F, Angheben A, et al. Strict adherence to malaria rapid test results might lead to a neglect of other dangerous diseases: a cost benefit analysis from Burkina Faso. Malar J. 2011;10:226.
9. Boadu NYA, Ansong D, Amuasi JH, Nguah SB, Arhin B, Somuah S, et al. A review of malaria rapid diagnostic tests (RDT) guideline implementation in a district hospital in ghana: Has rapid testing been prioritized? American journal of tropical medicine and hygiene. 2012;1):397.
10. Bottieau E, Gillet P, De Weggheleire A, Scheirlinck A, Stokx J, Das Dores Mosse C, et al. Treatment practices in patients with suspected malaria in Provincial Hospital of Tete, Mozambique. Trans R Soc Trop Med Hyg. 2013;107(3):176-82.
11. Boyce RM, Muiru A, Reyes R, Ntaro M, Mulogo E, Matte M, et al. Impact of rapid diagnostic tests for the diagnosis and treatment of malaria at a peripheral health facility in Western Uganda: an interrupted time series analysis. Malar J. 2015;14:203.
12. Boyce MR, O'Meara WP. Use of malaria RDTs in various health contexts across sub-Saharan Africa: a systematic review. BMC Public Health. 2017;17(1):470.
13. Brasseur P, Badiane M, Cisse M, Vaillant M, Olliaro PL. Changes in malaria prevalence and health provider's behavior towards fever with the introduction of act and RDT at peripheral health centre level in southwestern senegal (2000-2011). American journal of tropical medicine and hygiene. 2012;1):273.
14. Brigitte L. Interests in biological confirmation of malaria diagnosis in decision making for malaria treatment in the Democratic Republic of the Congo. American Journal of Tropical Medicine and Hygiene. 2020;103(5 SUPPL):162.
15. Bruxvoort K, Festo C, Thwing J, Thomson R, Kalolella A, Nchimbi H, et al. Over and under-use of artemisinin based combination therapy at public health facilities in three regions of Tanzania. American journal of tropical medicine and hygiene. 2011;1):194-5.
16. Bruxvoort K, Leurent B, Hopkins H. Impact of malaria rapid diagnostic tests on patient care: Results from the act consortium. American journal of tropical medicine and hygiene. 2015;93 (4 Supplement):464-5.
17. Bruxvoort KJ, Leurent B, Chandler CIR, Ansah EK, Baiden F, Bjorkman A, et al. The Impact of Introducing Malaria Rapid Diagnostic Tests on Fever Case Management: A Synthesis of Ten Studies from the ACT Consortium. Am J Trop Med Hyg. 2017;97(4):1170-9.
18. Burchett HE, Leurent B, Baiden F, Baltzell K, Bjorkman A, Bruxvoort K, et al. Improving prescribing practices with rapid diagnostic tests (RDTs): synthesis of 10 studies to explore reasons for variation in malaria RDT uptake and adherence. BMJ Open. 2017;7(3):e012973.
19. Catherine MS, DiLiberto D, Chandler C, Webb E, Othieno L, Nankya F, et al. Evaluating the impact of enhanced health facilitybased care for malaria and febrile illnesses in children in uganda: The act prime study. American journal of tropical medicine and hygiene. 2012;1):165.
20. Chanda P, Castillo-Riquelme M, Masiye F. Cost-effectiveness analysis of the available strategies for diagnosing malaria in outpatient clinics in Zambia. Cost Eff Resour Alloc. 2009;7:5.
21. Chandler CI, Webb EL, Maiteki-Sebuguzi C, Nayiga S, Nabirye C, DiLiberto DD, et al. The impact of an intervention to introduce malaria rapid diagnostic tests on fever case management in a high transmission setting in Uganda: A mixed-methods cluster-randomized trial (PRIME). PLoS One. 2017;12(3):e0170998.
22. Chilongola J, Msoka E, Juma A, Kajeguka DC, Semuva H, Kituma E, et al. Antibiotics prescription practices for provisional malaria cases in three hospitals in Moshi, Northern Tanzania. Tanzania Journal of Health Research. 2015;17(3):10.
23. Chinkhumba J, Skarbinski J, Chilima B, Campbell C, Ewing V, San Joaquin M, et al. Comparative field performance and adherence to test results of four malaria rapid diagnostic tests among febrile patients more than five years of age in Blantyre, Malawi. Malar J. 2010;9:209.
24. Chukwu LC, Agbasi PU, Unekwe PC, Onyema PU. Pattern of parasitological response and symptoms clearance in patients treated with artemether-lumefantrine combination in the treatment

of uncomplicated malaria in elele, a malaria endemic area in rivers state Nigeria. FASEB Journal Conference: Experimental Biology. 2017;31(1 Supplement 1).
25. Cohen JL, Saran I. Diagnostic testing and adherence to antimalarial medication: evidence from a randomized controlled trial in Uganda2015; (4 Supplement):[463-4 pp.]. Available from: http://onlinelibrary.wiley.com/o/cochrane/clcentral/articles/869/CN-01249869/frame.html.
26. D'Acremont V, Kahama-Maro J, Swai N, Mtasiwa D, Genton B, Lengeler C. Reduction of anti-malarial consumption after rapid diagnostic tests implementation in Dar es Salaam: a before-after and cluster randomized controlled study. Malar J. 2011;10:107.
27. d'Acremont V, Malila A, Swai N, Tillya R, Kahama-Maro J, Lengeler C, et al. Withholding antimalarials in febrile children who have a negative result for a rapid diagnostic test. Clin Infect Dis. 2010;51(5):506-11.
28. Das S, Jang IK, Misiuda KI, La Barre P. Evaluation of next generation infection detection tests for malaria elimination and eradication. American journal of tropical medicine and hygiene. 2015;93 (4 Supplement):254.
29. de Carsalade GY, Lam Kam R, Lepere JF, de Brettes A, Peyramond D. [Can the thick drop/smear examination for malaria be replaced by a rapid diagnostic test in first intention? The Mayotte experience]. Med Mal Infect. 2009;39(1):36-40.
30. Eliades J, Martin T, Davis K, Wun J, Mkomwa Z, Guindo B, et al. Improving health care worker performance in adherence to testing and test results for malaria in eight sub-saharan African countries. American Journal of Tropical Medicine and Hygiene. 2017;97 (5 Supplement 1):291-2.
31. Engo AB, Ntamabyaliro N, Nzolo DB, Lula YN, Tona GL. Drug use in the management of severe malaria in public health facilities in Democratic Republic of Congo. American Journal of Tropical Medicine and Hygiene. 2019;101(5 Supplement):80.
32. Festo C, Thomson R, Bruxvoort K, Johanes B, Nchimbi H, Kalolella A, et al. Access and targeting of malaria treatment: Assessing policy impact of the affordable medicines facility-malaria and roll out of parasitological diagnosis in three regions of tanzania. American journal of tropical medicine and hygiene. 2012;1):4.
33. Gerstl S, Dunkley S, Mukhtar A, De Smet M, Baker S, Maikere J. Assessment of two malaria rapid diagnostic tests in children under five years of age, with follow-up of false-positive pLDH test results, in a hyperendemic falciparum malaria area, Sierra Leone. Malar J. 2010;9:28.
34. Githinji SW, Oyando R, Malinga J, Ejersa W, Amin A, Ye Y. Changes in confirmed and clinical malaria cases reported through the DHIS 2 software platform in Kenya, 2011-2015. American Journal of Tropical Medicine and Hygiene. 2018;99 (4 Supplement):344.
35. Gitonga CW, Kihara JH, Njenga SM, Awuondo K, Noor AM, Snow RW, et al. Use of rapid diagnostic tests in malaria school surveys in Kenya: does their under-performance matter for planning malaria control? Am J Trop Med Hyg. 2012;87(6):1004-11.
36. Gupta P, Narang M, Gomber S, Saha R. Effect of quinine and artesunate combination therapy on platelet count of children with severe malaria. Paediatr Int Child Health. 2017;37(2):139-43.
37. Halliday KE, Mathanga D, Witek-McManus S, Mtali A, Ali D, Sande J, et al. Impact of school-based program of malaria diagnosis and treatment on school attendance in southern Malawi. American journal of tropical medicine and hygiene. 2014;1):381.
38. Halliday KE, Okello G, Turner EL, Njagi K, McHaro C, Kengo J, et al. Impact of intermittent screening and treatment for malaria among school children in Kenya: a cluster randomised trial. PLoS Med. 2014;11(1):e1001594.
39. Hamer DH, Brooks ET, Semrau K, Pilingana P, MacLeod WB, Siazeele K, et al. Quality and safety of integrated community case management of malaria using rapid diagnostic tests and pneumonia by community health workers. Pathog Glob Health. 2012;106(1):32-9.
40. Hamer DH, Ndhlovu M, Zurovac D, Fox M, Yeboah-Antwi K, Chanda P, et al. Improved diagnostic testing and malaria treatment practices in Zambia. JAMA. 2007;297(20):2227-31.

41. Harchut K, Standley C, Dobson A, Klaassen B, Rambaud-Althaus C, Althaus F, et al. Over-diagnosis of malaria by microscopy in the Kilombero Valley, Southern Tanzania: an evaluation of the utility and cost-effectiveness of rapid diagnostic tests. Malar J. 2013;12:159.
42. Harutyunyan V. Quality assurance of malaria rapid diagnostic tests (RDT) and its implication for clinical management of malaria. American journal of tropical medicine and hygiene. 2010;1):284.
43. Herlihy JM, D'Acremont V, Hay Burgess DC, Hamer DH. Diagnosis and Treatment of the Febrile Child. Washington DC: 2016 International Bank for Reconstruction and Development / The World Bank.; 2016.
44. Hopkins H, Bruxvoort KJ, Cairns ME, Chandler CI, Leurent B, Ansah EK, et al. Impact of introduction of rapid diagnostic tests for malaria on antibiotic prescribing: analysis of observational and randomised studies in public and private healthcare settings. BMJ. 2017;356:j1054.
45. Houze S, Boly MD, Le Bras J, Deloron P, Faucher JF. PfHRP2 and PfLDH antigen detection for monitoring the efficacy of artemisinin-based combination therapy (ACT) in the treatment of uncomplicated falciparum malaria. Malar J. 2009;8:211.
46. Hsiang MS, Ntshalintshali N, Kang Dufour MS, Dlamini N, Nhlabathi N, Vilakati S, et al. Active case-finding for malaria: A three-year national evaluation of optimal approaches to detect infections and hotspots through reactive case detection in the low transmission setting of Eswatini. Clinical infectious diseases : an official publication of the Infectious Diseases Society of America. 2019.
47. Huth PFB, Addo M, Daniel T, Groendahl B, Hokororo A, Koliopoulos P, et al. Extensive Antibiotic and Antimalarial Prescription Rate among Children with Acute Febrile Diseases in the Lake Victoria Region, Tanzania. Journal of tropical pediatrics. 2021;67(1).
48. Ige OK, Oladokun R. Challenges of malaria diagnosis in pediatric patients at a Nigerian hospital. American journal of tropical medicine and hygiene. 2014;1):78.
49. Ishengoma DS, Shayo A, Mandara C, Baraka V, Madebe R, Ngatunga D, et al. Performance of malaria rapid diagnostic tests for screening of patients to be enrolled in clinical trials and related studies. American journal of tropical medicine and hygiene. 2015;93 (4 Supplement):551.
50. Kalolella AB, Bruxvoort K, Thomson R, Festo C, Nchimbi H, Cairns M, et al. Addressing over- and under-diagnosis of malaria in Tanzania: An evaluation of large-scale implementation of malaria rapid diagnostic tests (MRDTS) in three regions with varying malaria epidemiology. American journal of tropical medicine and hygiene. 2012;1):100.
51. Kamau A, Mtanje G, Mataza C, Mwambingu G, Mturi N, Mohammed S, et al. Malaria infection, disease and mortality among children and adults on the coast of Kenya. Malaria journal. 2020;19(1):210.
52. Kapisi JA, Sserwanga A, Kigozi R, Opigo J, Yeka A, Kamya MR, et al. Antimalarial prescription practices at 21 public outpatient facilities located in regions of varying malaria endemicity in Uganda. American Journal of Tropical Medicine and Hygiene. 2017;97 (5 Supplement 1):528.
53. Kariuki S, Westercamp N, Owidhi M, Otieno K, Chebore W, Desai M, et al. Duration of malaria rapid diagnostic test (RDT) positivity following definitive antimalarial treatment among children in western Kenya. American journal of tropical medicine and hygiene. 2018;99(4):91-2.
54. Kiemde F, Mens P, Bonko A, Tahita M, Lompo P, Tinto H, et al. Impact of a malaria rapid diagnostic test detecting plasmodium falciparum-specific histidine-rich protein-2 (RDT-PFHRP2) on the management of febrile children under-5 years of age in a high seasonal malaria transmission area. American Journal of Tropical Medicine and Hygiene. 2017;97 (5 Supplement 1):405.
55. Kipanga PN, Omondi D, Mireji PO, Sawa P, Masiga DK, Villinger J. High-resolution melting analysis reveals low Plasmodium parasitaemia infections among microscopically negative febrile patients in western Kenya. Malar J. 2014;13:429.
56. Kitutu FE, Kalyango JN, Mayora C, Selling KE, Peterson S, Wamani H. Integrated community case management by drug sellers influences appropriate treatment of paediatric febrile illness in South Western Uganda: a quasi-experimental study. Malaria journal. 2017;16(1):425.

57. Kochar DK, Gupta V, Kochar A, Acharya J, Middha S, Sirohi P, et al. Comparison of quinine and rabeprazole with quinine monotherapy in the treatment of uncomplicated falciparum malaria. J Vector Borne Dis. 2010;47(3):140-4.
58. Kukula VA. Prescription practices among public and private health facilities for uncomplicated malaria in rural ghana. American journal of tropical medicine and hygiene. 2012;1):94.
59. Kumar JA, Kalule J, Elguero C, Dufort E, Tobin E. Correlation of malaria rapid diagnostic testing with clinical-based algorithm in a rural village in Uganda. American journal of tropical medicine and hygiene. 2011;1):395.
60. Lal S, Ndyomugenyi R, Magnussen P, Hansen KS, Alexander ND, Paintain L, et al. Referral Patterns of Community Health Workers Diagnosing and Treating Malaria: Cluster-Randomized Trials in Two Areas of High- and Low-Malaria Transmission in Southwestern Uganda. Am J Trop Med Hyg. 2016;95(6):1398-408.
61. Lal S, Ndyomugyenyi R, Hansen KS, Magnussen P, Clarke SE. Referral from community health workers: evidence from cluster randomized trials of mRDTs in two areas of high and low malaria transmission in Uganda2015; (4 Supplement):[360 p.]. Available from: http://onlinelibrary.wiley.com/o/cochrane/clcentral/articles/822/CN-01249822/frame.html.
62. Lawrence JJ, Ajayi T, Yeboah-Antwi K, MacLeod WB, Biemba G, Lunze K, et al. Clinical assessment of children with febrile Illness at health facilities in three districts in Southern Zambia. American journal of tropical medicine and hygiene. 2014;1):192.
63. Leurent B, Bruxvoort K, Grieve E, Hutchinson E, Reynolds J, Leslie T. Impact of malaria rapid diagnostic tests on care of febrile patients: Cross-project results from the act consortium. American journal of tropical medicine and hygiene. 2014;1):552.
64. Masanja IM, Selemani M, Amuri B, Kajungu D, Khatib R, Kachur SP, et al. Increased use of malaria rapid diagnostic tests improves targeting of anti-malarial treatment in rural Tanzania: implications for nationwide rollout of malaria rapid diagnostic tests. Malar J. 2012;11:221.
65. Mawili-Mboumba DP, Bouyou Akotet MK, Ngoungou EB, Kombila M. Evaluation of rapid diagnostic tests for malaria case management in Gabon. Diagn Microbiol Infect Dis. 2010;66(2):162-8.
66. Matangila JR, Lutumba P, Van Geertruyden JP. RDT are more cost effective to detect asymptomatic Plasmodium falciparum infection which is associated with anemia in pregnancy, Kinshasa, Democratic Republic of the Congo. International Journal of Infectious Diseases. 2014;21:5.
67. Mbonye AK, Buregyeya E, Rutebemberwa E, Lal S, Clarke SE, Hansen KS, et al. Treatment of Sick Children Seeking Care in the Private Health Sector in Uganda: A Cluster Randomized Trial. The American journal of tropical medicine and hygiene. 2020;102(3):658-66.
68. Mbonye AK, Magnussen P, Chandler CI, Hansen KS, Lal S, Cundill B, et al. Introducing rapid diagnostic tests for malaria into drug shops in Uganda: design and implementation of a cluster randomized trial. Trials. 2014;15:303.
69. Mbonye AK, Magnussen P, Hutchinson E, Hansen KS, Lal S, Clarke SE. Effects of introducing malaria rapid diagnostic tests in drug shops: Findings from the evaluation of a cluster randomised trial in Uganda2015:[16 p.]. Available from: http://onlinelibrary.wiley.com/o/cochrane/clcentral/articles/474/CN-01136474/frame.html.
70. Mbonye AK, Magnussen P, Lal S, Hansen KS, Cundill B, Chandler C, et al. A cluster randomized trial introducing rapid diagnostic tests into registered drug shops in Uganda: Impact on appropriate treatment of malaria. American journal of tropical medicine and hygiene. 2013;1):383.
71. Mbonye A, Magnussen P, Lal S, Hansen K, Cundill B, Chandler C, et al. A cluster randomised trial introducing rapid diagnostic tests into the private health sector in Uganda: Mpact on appropriate treatment of malaria. Tropical Medicine and International Health. 2013;18:74.
72. Mfuh KO, Achonduh-Atijegbe OA, Leke RG, Taylor DW, Nerurkar VR. Flipping the switch to accurately diagnose malaria: A comparison of clinical diagnosis, rapid diagnostic test (RDT),

microscopy and PCR in the diagnosis of malaria in Cameroon. American journal of tropical medicine and hygiene. 2015;93 (4 Supplement):550.
73. Mosha JF, Conteh L, Tediosi F, Gesase S, Bruce J, Chandramohan D, et al. Cost implications of improving malaria diagnosis: Findings from North-Eastern Tanzania. PLoS One. 2010;5 (1) (no pagination)(e8707).
74. Mubi M, Kakoko D, Ngasala B, Premji Z, Peterson S, Bjorkman A, et al. Malaria diagnosis and treatment practices following introduction of rapid diagnostic tests in Kibaha District, Coast Region, Tanzania. Malar J. 2013;12:293.
75. Munier A, Diallo A, Sokhna C, Chippaux JP. [Assessment of a rapid diagnostic test for malaria in rural health care facilities in Senegal]. Med Trop (Mars). 2009;69(5):496-500.
76. Ndyomugyenyi R, Hansen KS, Lal S, Chandler C, Mbonye AK, Magnussen P, et al. Introducing rapid diagnostic tests into community-based management of malaria: Evidence from a cluster-randomized trial in two areas of high and low transmission in Uganda. American journal of tropical medicine and hygiene. 2012;1):101-2.
77. Ndyomugyenyi R, Magnussen P, Lal S, Hansen KS, Chandler C, Clarke SE. Use of malaria rapid diagnostic test results among community medicine distributors in rural Ugandan communities: Impact on appropriate treatment. American journal of tropical medicine and hygiene. 2013;1):383.
78. Ndyomugyenyi R, Lal S, Hansen K, Chandler C, Magnussen P, Clarke S. Adherence to rapid diagnostic test results among community medicine distributors in rural Ugandan communities. Tropical Medicine and International Health. 2013;18:73-4.
79. Ndyomugyenyi R, Magnussen P, Hansen KS, Lal S, Chandler CI, Mbonye AK, et al. Improved targeting of antimalarial treatment in community-based management of malaria: Evidence from cluster-randomized trials in Uganda2014; (5 suppl. 1):[268 p.]. Available from: http://onlinelibrary.wiley.com/o/cochrane/clcentral/articles/347/CN-01056347/frame.html.
80. Newman MS, Rajan L. Are RDTs cost-effective and safe? A systematic review of accuracy in malaria diagnosis. American journal of tropical medicine and hygiene. 2009;1):53.
81. Njau JD, Kachur SP, McFarland D. A comparative cost-effective analysis study for adopting universal malaria rapid diagnostic tests (MRDT) in pediatric fevers across three sub-Saharan Africa countries. American journal of tropical medicine and hygiene. 2013;1):346.
82. Ojo AA, Maxwell K, Oresanya O, Adaji J, Hamade P, Tibenderana JK, et al. Health systems readiness and quality of inpatient malaria case-management in Kano State, Nigeria. Malaria journal. 2020;19(1):384.
83. Okolo CA, Erinle V, Oronsaye F, Temitope A, Ikhimioya U, Ismael Nana G, et al. Outcome of malaria rapid diagnostic test scale up on reducing presumptive diagnosis of malaria in challenging health settings: Evidence from 8 Nigerian States. American Journal of Tropical Medicine and Hygiene. 2020;103(5 SUPPL):184.
84. Osei-Kwakye K, Asante KP, Mahama E, Apanga S, Owusu R, Kwara E, et al. The benefits or otherwise of managing malaria cases with or without laboratory diagnosis: the experience in a district hospital in Ghana. PLoS One. 2013;8(3):e58107.
85. Otshudiema J, Embeke N, Hernandez F, Tchofa J, Modiri CM, Mwema FX. Scaling-up malaria rapid diagnostic tests and artemisinin-based combination therapy into integrated community case management sites: Results from two remote and low-resource settings in the democratic republic of Congo. American journal of tropical medicine and hygiene. 2013;1):382.
86. Oyeyemi OT, Ogunlade AF, Oyewole IO. Comparative assessment of microscopy and rapid diagnostic test (RDT) as malaria diagnostic tools. Research Journal of Parasitology. 2015;10(3):120-6.
87. Parikh R, Amole I, Tarpley M, Gbadero D, Davidson M, Vermund SH. Cost comparison of microscopy vs. empiric treatment for malaria in Southwestern Nigeria: a prospective study. Malar J. 2010;9:371.
88. Pinto MJ, Pereira NF, Rodrigues S, Kharangate NV, Verenkar MP. Rapid diagnosis of falciparum malaria by detection of Plasmodium falciparum HRP-2 antigen. J Assoc Physicians India. 1999;47(11):1076-8.

89. Portugal S, Tran TM, Ongoiba A, Bathily A, Li S, Doumbo S, et al. Treatment of Chronic Asymptomatic Plasmodium falciparum Infection Does Not Increase the Risk of Clinical Malaria Upon Reinfection. Clin Infect Dis. 2017;64(5):645-53.
90. Rantala AM, Taylor SM, Trottman PA, Luntamo M, Mbewe B, Maleta K, et al. Comparison of real-time PCR and microscopy for malaria parasite detection in Malawian pregnant women. Malaria journal. 2010;9 (1) (no pagination)(269).
91. Reyburn H, Mbatia R, Drakeley C, Carneiro I, Mwakasungula E, Mwerinde O, et al. Overdiagnosis of malaria in patients with severe febrile illness in Tanzania: a prospective study. Bmj. 2004;329(7476):1212.
92. Ruizendaal E, Schallig H, Traore M, Lompo P, Magloire NH, Traore O, et al. Malaria screening during pregnancy with RDTs performed by community health workers in Nanoro, Burkina Faso. Tropical medicine & international health. 2017;22:96-.
93. Salomao CA, Sacarlal J, Chilundo B, Gudo ES. Prescription practices for malaria in Mozambique: poor adherence to the national protocols for malaria treatment in 22 public health facilities. Malar J. 2015;14:483.
94. Sangoro O, Turner E, Simfukwe E, Miller JE, Moore SJ. A cluster-randomized controlled trial to assess the effectiveness of using 15% DEET topical repellent with long-lasting insecticidal nets (LLINs) compared to a placebo lotion on malaria transmission. Malar J. 2014;13:324.
95. Schrot-Sanyan S, Gaidot-Pagnier S, Abou-Bacar A, Sirima SB, Candolfi E. Malaria relevance and diagnosis in febrile Burkina Faso travellers: a prospective study. Malar J. 2013;12:270.
96. Searle KM, Pringle J, Hamapumbu H, Musonda M, Katowa B, Kobayashi T, et al. Evaluating the efficiency of reactive case detection to achieve malaria elimination in rural Southern Zambia using follow-up household visits and parasite genotyping. American Journal of Tropical Medicine and Hygiene. 2017;97 (5 Supplement 1):412.
97. Shakely D, Elfving K, Aydin-Schmidt B, Msellem MI, Morris U, Omar R, et al. The usefulness of rapid diagnostic tests in the new context of low malaria transmission in Zanzibar. PLoS One. 2013;8(9):e72912.
98. Srinivasan S, Moody AH, Chiodini PL. Comparison of blood-film microscopy, the OptiMAL dipstick, Rhodamine-123 fluorescence staining and PCR, for monitoring antimalarial treatment. Ann Trop Med Parasitol. 2000;94(3):227-32.
99. Swarthout TD, Counihan H, Senga RK, van den Broek I. Paracheck-Pf accuracy and recently treated Plasmodium falciparum infections: is there a risk of over-diagnosis? Malar J. 2007;6:58.
100. Tiruneh M, Gebregergs GB, Birhanu D. Determinants of delay in seeking treatment among malaria patients in Dera district, NorthWest Ethiopia: a case control study. African health sciences. 2018;18(3):552-9.
101. Uzochukwu BS, Obikeze EN, Onwujekwe OE, Onoka CA, Griffiths UK. Cost-effectiveness analysis of rapid diagnostic test, microscopy and syndromic approach in the diagnosis of malaria in Nigeria: implications for scaling-up deployment of ACT. Malar J. 2009;8:265.
102. Van Eijk AM. Subpatent malaria in pregnancy: A systematic review and individual patient data meta-analysis. American Journal of Tropical Medicine and Hygiene. 2019;101(5 Supplement):285-6.
103. Wang X, Zhong D, Otambo W, Atieli H, Yan G. Added value of ultra-sensitive rdt for malaria surveillance in endemic Africa in the context of rapidly changing epidemiology. American Journal of Tropical Medicine and Hygiene. 2020;103(5 SUPPL):318-9.
104. Wernsdorfer W. The use of rapid diagnostic tools - The end of traditional microscopic diagnosis of malaria? Tropical Medicine and International Health. 2009;14:28.
105. Williams HA, Causer L, Metta E, Malila A, O'Reilly T, Abdulla S, et al. Dispensary level pilot implementation of rapid diagnostic tests: an evaluation of RDT acceptance and usage by providers and patients--Tanzania, 2005. Malar J. 2008;7:239.
106. Wiseman V, Mangham-Jefferies L, Achonduh O, Drake T, Cundill B, Onwujekwe O, et al. Economic evaluation of interventions to improve health workers' practice in diagnosing and treating

uncomplicated malaria in Cameroon. American journal of tropical medicine and hygiene. 2014;1):268.
107. Wogu MN, Nduka FO. Evaluating Malaria Prevalence Using Clinical Diagnosis Compared with Microscopy and Rapid Diagnostic Tests in a Tertiary Healthcare Facility in Rivers State, Nigeria. Journal of tropical medicine. 2018;2018:3954717.
108. Wongsrichanalai C, Barcus MJ, Muth S, Sutamihardja A, Wernsdorfer WH. A review of malaria diagnostic tools: microscopy and rapid diagnostic test (RDT). Am J Trop Med Hyg. 2007;77(6 Suppl):119-27.
109. Yimam Y, Mohebali M, Abbaszadeh Afshar MJ. Comparison of diagnostic performance between conventional and ultrasensitive rapid diagnostic tests for diagnosis of malaria: A systematic review and meta-analysis. PloS one. 2022;17(2):e0263770.
110. Zuniga MA, Mejia RE, Sanchez AL, Sosa-Ochoa WH, Fontecha GA. Glucose-6-phosphate dehydrogenase deficiency among malaria patients of Honduras: a descriptive study of archival blood samples. Malar J. 2015;14:308.

Supplementary File 6: Characteristics of Included Studies

Author, year	Design and sample size	Loss to follow up	Country, Setting	Transmission, Parasite & Prevalence	Population	Intervention	Comparator	Patient-important outcomes
**Experimental studies**								
Ameyaw 2014 (21)	iRCT<br>N=240<br>I: 121<br>C:119	N=0	Ghana<br>Rural Healthcare (Government-owned hospital)	High transmission season<br>Predominant parasites; (*P. falciparum*, *P. vivax*, *P. ovale* & *P. malariae*)<br>Prevalence not specified	All children six months to 12 years with a tempt. above 37.5°C	RDT<br>Sens.=98.0 %<br>Spec.=83.3 %	Routine care	Symptom resolution on days two and five.<br>Mortality & prescription patterns at day five (endpoint)
Ansah 2010 (22)	iRCT<br>**Microscopy setting**<br>N=3811<br>I:1904<br>C: 1907<br>**Clinical setting**<br>N=3452<br>I:1725<br>C: 1727	N=175	Ghana<br>Rural Healthcare (Government-owned & private health centre)	Season not specified<br>Predominant parasite; (*P. falciparum*)<br>Prevalence not specified	All patients considered for malaria treatment by healthcare workers	RDT<br>**Microscopy setting:**<br>Sens.=86.9 %<br>Spec.=88.0 %<br>**Clinical setting:**<br>Sens.=93.1 %<br>Spec.=90.1 %	Microscopy & Presumptive diagnosis	Prescription patterns & mortality at day 28 days.

Ansah 2013 (23)	cRCT<br>**Microscopy setting:**<br>N=2000<br>I =1000<br>C= 1000<br>**Clinical setting:**<br>N=2000<br>I= 1000<br>C= 1000	Not specified	Ghana<br>Rural & urban Healthcare (Government-owned health centres)	Season not specified<br>Predominant parasite; (*P. falciparum*)<br>Prevalence not specified	All patients visiting the health facility and suspected of malaria.	RDT<br>**Microscopy setting:**<br>Sens.=87%<br>Spec.=88 %<br>**Clinical setting:**<br>Sens.=93%<br>Spec.=90 %	Microscopy & Presumptive diagnosis	Patient health cost at study endpoint<br>- Adherence to test results
Ansah 2015 (24)	cRCT<br>N=4817<br>I: 2719<br>C: 2098	N=214	Ghana<br>Rural & urban Community (Private pharmacies)	High & low transmission season<br>Predominant parasite; (*P. falciparum*)<br>Prevalence not specified	All clients reporting to a chemical shop complaining of fever or requesting an antimalarial	RDT<br>Sens.=96%<br>Spec.=70 %	Presumptive diagnosis	Prescription patterns during the study period & referral rate within day 28 after diagnosis
Baiden 2016 (25)	cRCT<br>N=3046<br>I: 1527<br>C:1519	N=760	Ghana<br>Rural Healthcare (Government-owned health centres)	High transmission season<br>Predominant parasite; (*P. falciparum*)<br>Prevalence not specified	All febrile children aged below 24 months at the first visit	RDT<br>Sens.=95%<br>Spec.=64 %	Presumptive diagnosis	Mortality & prescription patterns during the 24 months of the study period
Batwala 2011-a (26)	CECT<br>N=52116<br>I: 17637<br>C:16971 <presumptive arm> & 17508 microscopy arm>	Not specified	Uganda<br>Rural Healthcare (Government-owned health centres)	Low transmission season<br>Predominant parasite; (*P. falciparum*)<br>Prevalence not specified	All children aged between 3 & 59 months presenting at the study health centres with fever	RDT<br>Sens. & spec. not specified	Presumptive diagnosis & microscopy	Prescription patterns during the study period

Batwala 2011-b (27)	cRCT<br>N= 102087<br>I:46131<br>C:23884<presumptive arm> &<br>32072<microscopy arm>	Not specified	Uganda<br>Rural<br>Healthcare (Government-owned health centres)	High & low transmission season<br>Predominant parasite; (*P. falciparum*)<br>Prevalence not specified	All outpatients presenting at the study health centres with fever	RDT<br>Sens. & spec. not specified	Presumptive diagnosis & microscopy	Prescription patterns, time to treatment & time to diagnosis at the study endpoint
Bisoffi 2009 (28)	iRCT<br>N=2141<br>Dry season-813<br>I:388<br>C:425<br>Wet season-1282<br>I:636<br>C:646	N=74	Burkina Faso<br>Rural & urban<br>Healthcare (Government-owned health centres)	High & low transmission season<br>Predominant parasite; (*P. falciparum*)<br>Prevalence not specified	Participants aged six months and above and presenting with a temperature > 37.5°C	RDT<br>Sens.=52%<br>Spec.=99.5 %	Presumptive diagnosis	Symptom resolution, antimalarial & antibiotic prescription patterns & mortality at day four
Hansen 2017 (29)	cRCT<br>N=2000<br>I:1000<br>C:1000	Not specified	Uganda<br>Rural<br>Research (Villages within selected communities)	Moderate & low transmission season<br>Predominant parasite; (*P. falciparum*)<br>Prevalence not specified	All children under five presenting with fever to a CHW	RDT<br>Sens. & spec. not specified	Presumptive diagnosis	Patient health cost at study endpoint
Mbonye 2015 (30)	cRCT<br>N=15517<br>I:8672<br>C:6845	N=647	Uganda<br>Rural & peri-urban<br>Community (Private propriety)	High transmission season<br>Predominant parasite; (*P. falciparum*)<br>Prevalence not specified	All febrile patients presenting to retail drug stores	RDT<br>Sens.=91.7 %<br>Spec.=63.1 %	Presumptive diagnosis	Prescription patterns at the study endpoint

Mubi 2011 (31)	Randomised crossover trial<br>N=2930<br>I:1457<br>C:1473	N=61	Tanzania<br>Rural<br>Healthcare & community (Government-owned health dispensaries)	High transmission season<br>Predominant parasite; (Not specified)<br>Prevalence not specified	History of fever in the preceding 24 hours in patients above three months	RDT<br>Sens.=85.3 %<br>Spec.=59.8 %	Presumptive diagnosis	Prescription patterns, mortality & adherence to test results on days three, seven and twenty-eight after diagnosis
Mukanga 2012 (32)	cRCT<br>N=4216<br>I:2084<br>C:2132	N=199	Uganda, Burkina Faso, Ghana<br>Rural<br>Healthcare (Government-owned health units)	High, moderate & low transmission season<br>Predominant parasite; (*P. falciparum*)<br>Prevalence not specified	Children aged up to 59 months with measured or history of fever (last 24 hours)	RDT<br>Sens. & spec. not specified	Presumptive diagnosis	Symptom resolution & prescription patterns on days zero, three and seven of the study
Ndyomugyenyi 2016 (33)	cRCT<br>N=2575<br>Low season;<br>I: 403<br>C: 817<br>Moderate-high season;<br>I: 656<br>C:699	N=0	Uganda<br>Rural and urban<br>Healthcare (Government-owned health units)	High, moderate & low transmission season<br>Predominant parasite; (*P. falciparum*)<br>Prevalence= 50%	Febrile children aged under five years of age	RDT<br>Sens.=72.1 %<br>Spec.=83.3 %	Presumptive diagnosis	Prescription patterns & time to treatment during the study
Reyburn 2007 (34)	iRCT<br>N= 2416<br>I: 1202<br>C: 1214	N=19	Tanzania<br>Rural<br>Healthcare (Government-owned hospital)	High & low transmission season<br>Predominant parasite; (*P. falciparum*)<br>Prevalence not specified	A clinician's decision to request a malaria test in a patient of any age and sex	RDT<br>Sens.=95.4 %<br>Spec.=95.9 %	Microscopy	Prescription patterns & pre-treatment loss to follow-up at study endpoint

Yeboah-Antwi 2010 (35)	cRCT<br>N=3125<br>I:1017<br>C:2108	N=77	Zambia<br>Rural<br>Healthcare & community (Private non-profit hospitals and health posts)	High transmission season<br>Predominant parasite; (*P. falciparum*)<br>Prevalence not specified	Children aged six months to 5 years with fever or cough or difficult or fast breathing	RDT<br>Sens.=95%<br>Spec.=75 %	Presumptive diagnosis	Prescription patterns, symptom resolution & mortality on days five to seven of the study
**Quasi-experimental studies**								
Awor 2014 (36)	Pre-post<br>N= 943<br>Before RDT<br>N=163<br>I:80<br>C:83<br>After RDT<br>N=780<br>I:497<br>C:283	Not specified	Uganda<br>Rural<br>Community (Pharmacies)	High transmission season<br>Predominant parasite; (Not specified)<br>Prevalence= 60%	Children & their caretakers with a history of fever, cough or diarrhoea	RDT<br>Sens. & spec. not specified	Presumptive diagnosis	ACT & antibiotic prescription patterns after two months
Bruxvoort 2013 (37)	Pre-post<br>N=3456<br>I (post-RDT):1710<br>C (pre-RDT):1746	N=0	Tanzania<br>(Area not specified)<br>Healthcare (Government-owned hospitals, health centres & dispensaries)	Season not specified<br>Predominant parasite; (*P. falciparum*)<br>Prevalence= 18.6%, 17.4% & 0.5% in Mwanza, Mtwara & Mbeya respectively	Outpatients with fever or history of fever in the previous 48 hours	RDT<br>Sens.=91.3 %<br>Spec.=88 %	Microscopy	Antibiotic and ACT prescription patterns at the study endpoint

Ishengoma 2011 (38)	Pre-post<br>N=7397<br>I (post-RDT):18217<br>C (pre-RDT):5576	Not specified	Tanzania<br>Rural<br>Community (Villages)	Low transmission season<br>Predominant parasite; (*P. falciparum*)<br>Prevalence not specified	All febrile patients presenting to community health workers	RDT<br>Sens.=89.2 %<br>Spec.=74.3 %	Microscopy	Antimalarials prescription patterns at the study endpoint
Msellem 2009 (39)	Non-randomised crossover trial<br>N=1887<br>I:1005<br>C: 882	N=0	Tanzania<br>Rural<br>Healthcare (Government-owned health dispensary)	High transmission season<br>Predominant parasite; (*P. falciparum*)<br>Prevalence= 30%	All patients of all ages with a history of fever-last 48 hrs	RDT<br>Sens.=92%<br>Spec.=88 %	Presumptive diagnosis	Antimalarial and antibiotic prescription patterns after fourteen days & the rate of clinical re-attendance
Ukwaja 2010 (40)	Pre-post<br>N=100<br>I (post-RDT):50<br>C (pre-RDT):50	N=0	Nigeria<br>Urban<br>Healthcare (Government-owned health centre)	High transmission season<br>Predominant parasite; (*P. falciparum*)<br>Prevalence not specified	Children aged two months to 59 months with clinical malaria & pneumonia	RDT<br>Sens. & spec. not specified	Presumptive diagnosis	Risk of prescribing antimalarials, symptom resolution during follow-up and rates of clinical re-attendance at day five
**Observational studies**								
Bonful 2019 (41)	Analytical cross-sectional study<br>N=2519<br>I=1007<br>C= 1512	N=0	Ghana<br>Urban<br>Healthcare (Private non-profit hospital)	Season not specified<br>Predominant parasite; (Not specified)<br>Prevalence not specified	Febrile outpatients presenting with a temperature of 37.5°C	RDT<br>Sens. & spec. not specified	Presumptive diagnosis	Inappropriate ACT prescription patterns among patients at the study endpoint

Bonko 2019 (42)	Analytical cross-sectional study<br>N=2195<br>I= 1098<br>C=1097	N=0	Burkina Faso<br>Rural<br>Healthcare (Government-owned hospital & health centre)	High transmission season<br>Predominant parasite; (*P. falciparum*)<br>Prevalence not specified	All under five yrs. children presenting with a temperature of 37.5°C & above	RDT<br>Sens. & spec.>95%	Microscopy	Risk of antimalarials & antibiotics prescription among RDT positive & negative patients at the study endpoint<br>Adherence to test results at the study endpoint
Ikwuobe 2013 (43)	Analytical cross-sectional study<br>N=1226<br>I:619<br>C:607	Not specified	Nigeria<br>Rural & urban<br>Community (Private propriety pharmacy)	High transmission season<br>Predominant parasite; (*P. falciparum*)<br>Prevalence not specified	Patients with symptoms of uncomplicated malaria>10 years	RDT<br>Sens.=99.7 %<br>Spec.=99.5 %	Presumptive diagnosis	Antimalarials prescription patterns at the study endpoint
Yukich 2010 (44)	Cohort<br>N= 259<br>I: 122<br>C:137	N=0	Tanzania<br>Rural & peri-urban<br>Healthcare (Government-owned hospital & health dispensary)	Season not specified<br>Predominant parasite; (*P. falciparum*)<br>Prevalence=<10 %	All patients with uncomplicated malaria at the first visit	RDT<br>Sens. & spec. not specified	Microscopy	Patient health costs after one week

iRCT-Individual Randomized Controlled Trials; cRCT-Cluster Randomized Controlled Trials; N-Total number of patients included, I-Number of patients in the intervention arm (RDT); C-Number of patients in the comparator arm (Clinical diagnosis or microscopy); Sens.-Sensitivity; Spec.-Specificity; CHW-Community Health Worker; ACT-Artemisinin-based Combination Therapy, Quasi-experimental studies: Non-randomized studies of intervention.

Supplementary File 7: Results of the patient-important outcome measures

Author Year	Results of the measured patient-important outcomes		
	**Diagnostic impact**	**Therapeutic impact**	**Patient-outcome impact**
		**Experimental studies**	
**Ameyaw 2014 (21)**	Not reported	Not reported	Symptom resolution;<br>**I: 120/121 (99.2%) C: 96/119 (80.7%), P<0.001**<br>Mortality;<br>I: 0/121 (0.0%) C: 1/119 (0.8%), P=0.496
**Ansah 2010 (22)**	Not reported	Prescription patterns;<br>*Wrongly treated with antimalarials*<br>Microscopy setting:<br>I: 722/1400 (51.6%), C: 764/1389 (55%), OR=0.87 [95% CI 0.71-1.1] P=0.16<br>Clinical setting:<br>**I: 578/1072 (53.9%), C:982/1090 (90.1%), OR=0.12 [95% CI 0.04-0.38] P=0.001**	Mortality;<br>Microscopy setting:<br>I: 4/1904 (0.0%) C: 2/1907 (0.1%)<br>Clinical setting:<br>I: 0/1725 (0.0%) C: 4/1727 (0.2%)
**Ansah 2013 (23)**	Not reported	Adherence to test result<br>Microscopy setting:<br>I: 54%, C: 51%<br>Clinical setting:<br>I: 51%, C: 50%	Health costs (Ghana cedis);<br>Microscopy setting:<br>I: 6849 GHS C: 6892 GHS<br>Clinical setting:<br>I: 6924 GHS C: 7677 GHS
**Ansah 2015 (24)**	Not reported	Prescription patterns:<br>*Prescription of antimalarial in negative cases;*<br>**I: 32%, C: 88%, P<0.0001, Risk ratio: 0.41 95% CI: (0.29-0.58)**<br>*Prescription of antibiotics in patients with negative cases;*<br>I: 6/1854 (0.3%) C: 1/1570 (0.1%)	Referrals<br>**Less than 1.5% (13/1071) of all the slide-positive clients were referred to another health facility, with the majority being from the rapid diagnostic test arm (p=0.024)**
**Baiden 2016 (25)**	Not reported	Prescription patterns:<br>*ACT;*<br>**I: 72.3% C: 80.8% P=0.02**<br>*Antibiotic;*<br>I: 54.8% C: 56.2% P=0.78	Mortality;<br>I: 15/1527 (1.0%) C: 21/1519 (1.4%), P=0.31

**Batwala 2011 (26)-a**	Not reported	Prescription of antibiotics;<br>I: 810/17637 (56.2%) 95% CI: (56.3-58.7), C: <presumptive> 7040/16971 (41.5%) 95% CI: (40.7-42.2), <microscopy> 273/ 17508 (17.6%) 95% CI: (15.7-19.5)	Not reported
**Batwala 2011 (27)-b**	Time to diagnosis:<br>*Mean patient time in minutes (mean/95%CI):*<br>Overall: 62.4 [54.6-70.2], I: 37.5[32.8-42.3], C <Microscopy>: 123.9 [105.9-142.0], C<Presumptive>: N/A	Antimalarial amongst patients not tested as randomized;<br>I: 870/1566 (60.3%) 95% CI: (57.9-62.8), C: <presumptive> 16931/23884 (99.7%) 95% CI: (99.6-99.8), <microscopy> 6266/ 12527(97.5%) 95% CI: (96.5-98.5)<br>Time to treatment;<br>*Mean patient time in minutes (mean/95%CI):*<br>[Overall: 133.7, 95% CI: (126.0-141.3), I: 109.2, 95% CI: (98.3-120.1), Microscopy: 156.1, 95% CI: (141.4-170.9)	Not reported
**Bisoffi 2009 (28)**	Not reported	Prescription patterns:<br>*Antimalarial:*<br>Dry Season; I: 340/404 (84.2%), C: 359/448 (80.1%), P=0.13<br>Wet season; I: 605/654 (92.5%), C: 610/663 (92.0%), P=0.73<br>*Antibiotic:*<br>Dry season; I: 229/404 (56.7%), C: 275/448 (61.4%), P=0.16<br>Wet season; I: 331/654 (50.6%), C: 334/663 (50.3%), P=0.93	Symptom resolution:<br>Dry Season I:32/388 (8.2%), C:35/425(8.2%), P=0.99<br>Wet Season I:25/636 (3.9%), C:34/646(3.7%), P=0.83<br>Mortality:<br>Dry Season I:4/388 (1.0%), C:3/425(0.7%), P=0.71<br>Wet Season I:1/636 (0.15%), C:1/646(0.15%), P=1
**Hansen 2017 (29)**	Not reported	Not reported	Patient health cost:<br>*Per 1000 children in U.S. dollars;*<br>I: $ 33, C: $ 27
**Mbonye 2015 (30)**	Not reported	Prescription patterns:<br>*Patients given appropriate treatment:*<br>**Cluster mean (95% CI) I: 52.8 (45.9-59.7), C: 26.8 (19.5-34.2), P<0.001, Risk difference: 25.2% (12.3-38.0)**	Not reported

**Mubi 2011 (31)**	Not reported	Prescription patterns:<br>*ACT:*<br>**I: 775/1457 (53.2%) C: 1422/1473 (96.5%) OR) 0.039, 95% CI 0.029–0.053)**<br>Adherence to test results:<br>**I: 97.4% and CD arm=99.3% (OR 3.3, 95% CI 1.5–7.7)**	Mortality:<br>4 patients died during the trial- 3children below 5 years within three days and 1 adult within seven days<br>Referrals:<br>More patients were referred on inclusion day during RDT weeks (10.0%) compared to CD weeks (1.6%)
**Mukanga 2012 (32)**	Not reported	Prescription pattern:<br>*Positive cases that did not receive ACTs;*<br>I: 1/1740 (0.05%), C: 17/344 (4.9%)	Symptom resolution:<br>*Fever clearance rate:*<br>I: 99.4% 95% CI: (98.8, 99.99), C: 99.0% 95% CI: (98.7, 99.4) OR=0.64, 95% CI: (0.28, 1.49).
**Ndyomugyenyi 2016 (33)**	Not reported	Time to treatment:<br>*Frequency of patients treated within 24 hours of onset of symptoms:*<br>Low season; **I: 287 (72.1%), C: 49 (6.0%) OR: 40.3 95% CI (28.1-57.9), P<0.001)**<br>Moderate-high season; **I: 433 (67.0%), C: 195 (28.1%) OR: 5.92 95% CI (4.15-8.45), P<0.001)**<br>Prescription patterns:<br>*ACTs in negative cases:*<br>Low season; **I: 22 (5.81%), C: 749 (97.2%) OR: 0.00022 95% CI (0.00004-0.00125), P=0.002)**<br>Moderate-high season; I: 67 (16.4%), C: 484 (99.2%) OR: 0.0013 95% CI (-0.0004-0.0039), P<0.001)	Not reported
**Reyburn 2007 (34)**	Not reported	Prescription pattern:<br>*Correct antimalarial prescription;*<br>I: 616/1193 (51.6%) C: 606/1204 (50.3%) OR=1.05, 95% CI= 0.90-1.12, P=0.524<br>Pre-treatment loss to follow-up:<br>I: 9/1202 (0.75%), C: 10/1214 (0.82%)	Not reported
**Yeboah-Antwi 2010 (35)**	Not reported	Prescription pattern:<br>*Antimalarial;*<br>**I: 265/963 (27.5%), C: 2066/2084 (99.1%) RR=0.23, 95% CI= 0.14-0.38**<br>*Antibiotic;*	Symptom resolution:<br>*Hospitalized:*<br>I: 4/1017 (0.4%), C: 14/2108 (0.7%) RR= 0.25 95% CI: 0.04-1.15<br>*Treatment failure:*

		**I: 247/362 (68.2%) C: 22/203 (13.3%) RR=5.32, 95% CI= 2.19-8.94**	I: 95/1017 (9.3%), C: 211/2108 (10.0%) RR= 0.68 95% CI: 0.39-1.19<br>Mortality:<br>I: 2/1017 (0.19%), C: 1/2108 (0.04%)
**Quasi-experimental studies**			
**Awor 2014 (36)**	Not reported	Prescription patterns:<br>*Before (Pre)*<br>*ACT;*<br>I: 12/74 (16.2%) C: 27/71 (38.0%)<br>*Antibiotic;*<br>I: 54/80 (65.1%) C: 36/86 (45.0%) P=0.78<br><br>*After (Post)*<br>*ACT;*<br>**I: 393/487 (80.7%) C: 113/275 (41.1%) PR (95% CI): 4.2 (1.9–9.4)**<br>*Antibiotic;*<br>**I: 298/497 (60.0%) C: 208/2 83 (73.5%) PR (95% CI): 0.82(0.69–0.97)**	Not reported
**Bruxvoort 2013 (37)**	Not reported	Prescription patterns:<br>*Percentage of patients obtaining ACT before RDT*<br>**I: 39.9%, C: 21.3%, P<0.0001)**<br>*Percentage of patients obtaining ACT after*<br>**I: 31.2% C: 48.5%, P<0.0001)**	Not reported
**Ishengoma 2011 (38)**	Not reported	Prescription patterns:<br>*Antimalarials;*<br>I: 32.1% C: 98.9% in cases aged more than or equal to 5 years	Not reported
**Msellem 2009 (39)**	Not reported	Prescription patterns:<br>*Antimalarials;*<br>**I: 361/1005 (36%) C: 752/882 (85%) (OR: 0.04, 95% CI: 0.03–0.05, P<0.001)**<br>*Antibiotics;*<br>**I: 372/1005 (37%) C: 235/882 (27%) (OR: 1.8, 95% CI: 1.5–2.2, P<0.001)**	Clinical re-attendance:<br>**I: 25/1005 (2.5%) C: 3/882 (4.9%) (OR: 0.5, 95% CI: 0.3–0.9, P<0.005)**<br>Health costs:<br>*Average cost per patient (U.S. dollar;)*<br>I: USD 2.47 C: USD 2.37

**Ukwaja 2010** (40)	Not reported	Risk of antimalarial prescription:<br>**I: 48% C: 100% (RR 2.08, 95% CI: 1.56 to 2.78, P<0.001)**	Symptom resolution:<br>I: 47/50 (94%) C: 49/50 (98%) P=0.31)<br>Clinical re-attendance:<br>I: 9/50 (18%) C: 3/50 (6%) P=0.065)
**Observational studies**			
**Bonful 2019** (41)	Not reported	Inappropriate ACT prescription:<br>*Patients with negative test results treated using ACTs;*<br>145/679 (21.4%)<br>*Patients treated presumptively using ACTs;*<br>646/1512 (42.7%)	Not reported
**Bonko 2019** (42)	Not reported	Prescription patterns:<br>*Antimalarial;*<br>**I: 804/1098 (73.2%) C: 803/1097 (75.6%) (R.R. = 7.74 95% CI: 5.69-10.51, P < 0.0001)**<br>*Antibiotic:*<br>**I: 856/1098 (77.9%) C: 856/1097 (78%) (R.R. = 3.57 95% CI: 2.37-5.38, P < 0.0001)**<br>Adherence to test results;<br>I: 762/1020 (74.7%) C: 258/1020 (25.3%)	Not reported
**Ikwuobe 2013** (43)	Not reported	Prescription patterns:<br>Antimalarial in the negative cases: 276/535 (51.6%)<br>Antimalarial in the positive cases:<br>84/84 (100%)	Not reported
**Yukich 2010** (44)	Not reported	Not specified	Patient health cost<br>*Total mean cost per patient (Tanzanian shilling& U.S. dollar):*<br>I: $1.02 (95% CI; 0.76-1.36), TSh 1,247, SD: 2,021, C: $1.33 (95% CI; 0.99-1.77), TSh 1,630, SD: 1,826]; P= 0.033

RR-Risk ratio; PR-Prevalence ratio

*Results in **bold** denote outcome measures that were reported to be statistically significant*

Quasi-experimental studies: Non-randomized studies of intervention

Supplementary file 8: Summary of the methodological quality of controlled intervention studies

Study identification	Randomisation description	Method of randomisation	Allocation concealment	Participant/provider blinding	Blinding of outcome assessor	Similarity of groups (baseline)	<20% overall drop-out rate	<15% differential drop-out rate	Adherence to study protocols	Similar background treatment	Valid outcome measurement	Sample size justification	Prespecified outcomes	Intention to treat analysis	Overall quality
**Experimental Studies**															
**Ameyaw 2014**	Yes	Yes	Yes	N/A	N/A	Yes	Yes	Yes	Yes	Yes	Yes	Yes	Yes	Yes	Good
**Ansah 2010**	Yes	Yes	Yes	N/A	N/A	Yes	Yes	Yes	C/D	Yes	Yes	Yes	Yes	Yes	Good
**Ansah 2013**	No	Yes	Yes	N/A	N/A	N/R	N/R	N/R	Yes	Yes	Yes	Yes	Yes	Yes	Fair
**Ansah 2015**	Yes	Yes	No	N/A	N/A	Yes	Yes	Yes	Yes	No	Yes	Yes	Yes	Yes	Good
**Baiden 2016**	Yes	Yes	N/R	N/A	N/A	Yes	No	Yes	Yes	Yes	Yes	Yes	No	Yes	Good
**Batwala 2011**	Yes	Yes	N/R	N/A	N/A	Yes	N/R	N/R	Yes	Yes	Yes	No	Yes	Yes	Fair
**Batwala 2011**	Yes	C/D	C/D	N/A	N/A	Yes	Yes	Yes	No	Yes	Yes	N/R	N/R	Yes	Fair
**Bisoffi 2009**	Yes	Yes	N/R	N/A	N/A	Yes	Yes	Yes	No	Yes	Yes	Yes	Yes	Yes	Good
**Hansen 2017**	Yes	N/R	C/D	N/A	N/A	Yes	N/R	N/R	Yes	Yes	Yes	Yes	Yes	Yes	Fair
**Mbonye 2015**	Yes	Yes	C/D	N/A	N/A	N/R	Yes	Yes	No	Yes	Yes	Yes	Yes	Yes	Good
**Mubi 2011**	Yes	Yes	Yes	N/A	N/A	Yes	Yes	C/D	Yes	C/D	Yes	Yes	Yes	Yes	Good
**Mukanga 2012**	Yes	No	N/R	N/A	N/A	Yes	Yes	Yes	Yes	Yes	Yes	Yes	Yes	Yes	Good

**Ndyomugyenyi 2016**	Yes	Yes	N/R	N/A	N/A	Yes	Yes	Yes	Yes	Yes	Yes	Yes	Yes	Yes	Good
**Reyburn 2007**	Yes	Yes	Yes	N/A	N/A	Yes	Yes	Yes	Yes	Yes	Yes	Yes	Yes	Yes	Good
**Yeboah-Antwi 2010**	Yes	Yes	N/R	N/A	N/A	N/R	Yes	Yes	Yes	Yes	Yes	Yes	Yes	Yes	Good
**Quasi-Experimental Study Designs**															
**Awor 2014**	N/A	N/A	N/A	N/A	N/A	Yes	Yes	Yes	Yes	Yes	Yes	Yes	Yes	N/A	Good
**Bruxvoort 2013**	N/A	N/A	N/A	N/A	N/A	Yes	N/R	N/R	No	Yes	Yes	Yes	Yes	N/A	Fair
**Ishengoma 2011**	N/A	N/A	N/A	N/A	N/A	Yes	C/D	C/D	No	Yes	Yes	No	Yes	N/A	Fair
**Msellem 2009**	N/A	N/A	N/A	N/A	N/A	Yes	Yes	Yes	Yes	Yes	Yes	Yes	Yes	N/A	Good
**Ukwaja 2010**	N/A	N/A	N/A	N/A	N/A	Yes	Yes	Yes	Yes	Yes	Yes	No	Yes	N/A	Good

Supplementary file 9: Summary of the methodological quality of observational studies

Criteria	Bonful 2019	Bonko 2019	Ikwuobe 2013	Yuckich 2010
Research question	Yes	Yes	Yes	Yes
Detailed description of study population	Yes	Yes	Yes	Yes
Over 50% participation rate	C/D	Yes	Yes	Yes
Sampling of subjects from the same population	Yes	Yes	Yes	Yes
Sample size calculation & justification	Yes	No	Yes	Yes
Exposure measurement preceding outcome	N/A	N/A	N/A	Yes
Sufficient time frame	N/A	N/A	N/A	Yes
Measurement of exposure at different levels	N/A	N/A	N/A	N/A
Valid & reliable exposure measurement	C/D	Yes	Yes	Yes
Multiple exposure assessments	Yes	Yes	No	Yes
Valid & reliable outcome measurement	Yes	Yes	Yes	Yes
Blinding of outcome assessor	C/D	No	C/D	NR
Less than 20% loss to follow-up at endpoint	Yes	Yes	Yes	Yes
Confounder adjustment	Yes	No	Yes	Yes
**Overall quality**	Fair	Fair	Good	Good

For observational studies, except for Yuckich et al. which was a cohort study, the remaining three studies were cross-sectional. Therefore, items regarding exposure measurement preceding outcome and sufficient time frame were not applicable for cross-sectional studies. Management of exposure at different levels was not applicable to all observational studies because mRDTs were performed at a time.
For experimental studies blinding was not necessary given the nature. Of the quasi-experimental studies details on randomization were not applicable due to the nature of the design.
