# Supplementary material for: Evaluating the impact of malaria rapid diagnostic tests on patient-important outcomes in sub-Saharan Africa: a systematic review of study methods to guide effective implementation
Source: BMJ Open. 2024 Sep 10;14(9):e077361. doi: 10.1136/bmjopen-2023-077361 (PMC11409401; doi:10.1136/bmjopen-2023-077361)
Supplement: Supplementary file 3 [file bmjopen-2023-077361.reviewer_comments.pdf]

PEER REVIEW HISTORY

BMJ Open publishes all reviews undertaken for accepted manuscripts. Reviewers are asked to complete a checklist review form and are provided with free text boxes to elaborate on their assessment. These free text comments are reproduced below.

ARTICLE DETAILS

**TITLE (PROVISIONAL)**	EVALUATING THE IMPACT OF MALARIA RAPID DIAGNOSTIC TESTS (mRDTS) ON PATIENT-IMPORTANT OUTCOMES IN SUB-SAHARAN AFRICA: A SYSTEMATIC REVIEW OF STUDY METHODS TO GUIDE EFFECTIVE IMPLEMENTATION
**AUTHORS**	Otieno, Jenifer; Were, Lisa M.; Sagam, Caleb; Kariuki, Simon; Ochodo, Eleanor

VERSION 1 - REVIEW

**REVIEWER NAME**	Banek, Kristin
**REVIEWER AFFILIATION**	The University of North Carolina at Chapel Hill
**REVIEWER CONFLICT OF INTEREST**	None
**DATE REVIEW RETURNED**	30-Oct-2023

**GENERAL COMMENTS**	This manuscript describes the findings of a systematic review to identify studies reporting patient-important outcomes when assessing mRDT effectiveness. The authors suggest that effectiveness studies incorporate process evaluations to capture contextual and patient-related factors that may impact the design.<br>The strengths of this manuscript include:<br>• The paper is well-organized and justified.<br>• The methods are clear and could be repeated.<br>• The Tables, Figures, and supplements illustrate the review findings, making them easy to understand.<br><br>While the idea around this paper is good, I think the execution needs more work, which prevents a recommendation for it to move forward to publication at this time. Below, I list major issues that should be addressed, and a number of minor issues which could be considered.<br><br>Major<br>1. My first point is about the point of the review. It is unclear if the authors are trying to make the point that process evaluations should be included and are not often included. Or are the authors saying contextual and patient-important (often secondary) outcomes should be collected alongside effectiveness outcomes to better understand how to implement the intervention (mRDTs)? I think this is the main weakness of this paper, as the main objective is not clear.<br><br>2. While the paper aimed to identify patient-important outcomes and report the findings, I feel most readers would also be interested in the number of studies that lacked this. I know it is listed in the supplements, but I think it would give the issue more depth to frame the magnitude of the issue.<br><br>3. It seems some of the studies may have had patient-important

	outcomes that were not considered. One such example is the Gerstle et al. study in Sierra Leone, that shows an impact of referral completion. Referral can be life-saving and, therefore, wouldn't it be considered a patient-important outcome?<br><br>4. The domains and themes are clear, but a Main theme does not seem to emerge in the data. It seems that certain domains may be impacted more rather than the specific themes. Most themes only cite 1-3 papers for that theme. Consider listing the domains and themes as a table rather than just a long list in the text. Then, write about each domain in the test, citing the table.<br><br>5. Overall, the balance and content of the tables/figures/supplements effectively communicated the research results.<br>a. Figures 2/3 are a nice way to show quality, but they could use additional definitions. It is not exactly clear what each component includes. I believe some additional detail is in the supplement, but this could be presented more clearly.<br>b. Also, it would be good to use parallel language. I believe you have included semi-experimental in Table 2, but this is not immediately obvious.<br>c. Figure 3. There are items that are yes and not applicable rather than dichotomous, which is confusing. Consider grouping similar studies together in different quality figures, as it seems this may be what is causing the confusion.<br>d. The supplement tables contain a lot of information. A better description or key definitions could be at the bottom of the tables to ensure they can stand alone.<br><br>Minor<br>1. Page 7, line 19 remove the word "cross-sectional" as all studies not "evaluating the tests' impact on patient-important outcomes and modelling studies" were excluded, not just those that were cross-sectional<br>2. Page 11, line 10. Did any studies use PCR as a comparator? If not, state this was the case since PCR is listed as an inclusion criteria.<br>3. Studies' geographic distribution is important and could be discussed more. Most of the studies listed are from the same few countries. Since the idea is that context matters and that it should be measured, the point should be made that not only should process evaluations be performed, but that they should be performed in a variety of contexts.<br>4. Limitations of the review were not discussed. Consider addressing this.

**REVIEWER NAME**	Danwang, Celestin
**REVIEWER AFFILIATION**	Universite Libre de Bruxelles, School Of Public Health
**REVIEWER CONFLICT OF INTEREST**	None
**DATE REVIEW RETURNED**	15-Dec-2023

**GENERAL COMMENTS**	This is an important systematic review evaluating the methodology and implementation challenges of studies assessing the impact of malaria rapid diagnostic tests (mRDTs) on patient-important outcomes in sub-Saharan Africa. The topic is highly relevant given the widespread use of mRDTs and need to understand their real-

	world impact. The manuscript is clearly written and provides helpful insights. I have outlined some suggestions below to further strengthen the manuscript:<br><br>Major comments:<br><br>- The rationale behind including only studies published in English should be clearly justified. Given the geographic focus on sub-Saharan Africa, were relevant studies published in French or Portuguese excluded? This could limit generalizability.<br><br>- Along similar lines, The studies found seem to come from a limited number of countries. Do you think these results can be generalized to all Sub-Saharan Africa?<br><br>- Some further clarification on the definitions/categorization of patient-important outcomes would be helpful, or reference to a framework used. For example, are measures considered direct or indirect effects?<br><br>- The quality assessment revealed some key limitations of primary studies included. It would be useful to discuss how this could have biased the results or influenced the validity of findings.<br><br>- The implementation concerns seem highly relevant but could be structured or themed in a more meaningful way. Is there an existing framework the challenges could be mapped to? This would better highlight the most pressing issues.<br><br>- The implications for policy, practice and research could be expanded. In particular, more details on how the methodology of future mRDT impact studies could be improved would further add value.<br><br>Minor comments:<br>- Explain acronyms before first use (eg NMCPs).<br>- The objective should specify focus is on methodological evaluations.<br><br><br>Overall this an insightful, methodologically-focused systematic review. Addressing the suggestions above would further improve clarity and impact.

VERSION 1 – AUTHOR RESPONSE

Reviewer 1

Major Comments

My first point is about the point of the review. It is unclear if the authors are trying to make the point that process evaluations should be included and are not often included. Or are the authors saying contextual and patient-important (often secondary) outcomes should be collected alongside effectiveness outcomes to better understand how to implement the intervention (mRDTs)? I think this is the main weakness of this paper, as the main objective is not clear. Agreed and thank you for this insightful comment. We have revised the section on implications for practice, policy, and future research to depict: “…Patient-important outcomes should be measured alongside process evaluations to better understand how to implement mRDTs.” See pages 34-35.

While the paper aimed to identify patient-important outcomes and report the findings, I feel most

readers would also be interested in the number of studies that lacked this. I know it is listed in the supplements, but I think it would give the issue more depth to frame the magnitude of the issue. Agreed. While the focus of this paper is on studies measuring at least one patient-important outcome, the number of studies that lacked this is presented in Figure 1: PRISMA 2020 flow chart.
It seems some of the studies may have heard patient-important outcomes that were not considered. One such example is Gerstle et al. study in Sierra Leone, that shows an impact of referral completion. Referral can be life-saving and, therefore, wouldn't it be considered a patient-important outcome? Partially agreed. Although our search was comprehensive including both published and grey literature, we may have missed out on some studies, but regarding Gerstle et al. study in Sierra Leone, I found this study; Assessment of two malaria rapid diagnostic tests in children under five years of age, with follow-up of false-positive pLDH test results, in a hyperendemic falciparum malaria area, Sierra Leone - PubMed (nih.gov). Is it the one you were referring to? If yes, upon assessment, the study only reported on diagnostic accuracy measures.
The domains and themes are clear, but a Main theme does not seem to emerge in the data. It seems that certain domains may be impacted more rather than the specific themes. Most themes only cite 1-3 papers for that theme. Consider listing the domains and themes as a table rather than just a long list in the text. Then write about each domain in the test, citing the table. Agreed. Thank you for this insightful comment. We have revised the section to include a table and a brief text explaining the most emerging themes and domains of the SURE framework. See page 29.
Overall, the balance and content of the tables/figures/supplements effectively communicated the research results.
a. Figures 2/3 are a nice way to show quality, but they could use additional definitions. It is not exactly clear what each component includes. I believe some additional detail is in the supplement, but this could be presented more clearly.
b. Also, it would be good to use parallel language. I believe you have included semi-experimental in Table 2, but this is not immediately obvious.
c. Figure 3. There are items that are yes and not applicable rather than dichotomous, which is confusing. Consider grouping similar studies together in different quality figures, as it seems this may be what is causing the confusion.
d. The supplement tables contain a lot of information. A better description or key definitions could be at the bottom of the tables to ensure they can stand alone.
Agreed and thank you.
a). We have included definitions to clarify the Figures 2/3 in text.
b). We have also defined the term-quasi experimental study as used in Table 2.
c). We used the NIH'S quality assessment tool in which one tool is applicable for two study designs. Figure 3 illustrates the findings of both cross-sectional and cohort studies. Also given the journal's restriction on the number of tables/figures we are not able to present the quality assessment findings for each design separately. We have included descriptions pertaining to this below the figure.
d). We have included key definitions to the supplement tables.

Minor Comments
Page 6, line 19 remove the word "cross-sectional" as all studies not "evaluating the tests' impact on patient-important outcomes and modelling studies" were excluded, not just those that were cross-sectional. Agreed and thank you for noticing this. We have rectified the section to read: "We excluded studies, which only provided test accuracy statistics without evaluating the tests' impact on patient-important outcomes and modelling studies". See page 6.
Page 11, line 10. Did any studies use PCR as a comparator? If not, state this was the case since PCR is listed as an inclusion criterion. Thank you for this inquiry, none of the studies which we had included used PCR as a comparator. We have included this information in the results section. See page 10.
Studies' geographic distribution is important and could be discussed more. Most of the studies listed are from the same few countries. Since the idea is that context matters and it should be measured,

the point should be made that not only should process evaluations be performed, but they should be performed in a variety of contexts. Agreed and thank you. We have revised our statement to show that process evaluations should be performed in a variety of contexts. See page 32.
Limitations of this review were not discussed. Consider addressing this. Partially agreed. Although we had included in the discussion that our study was limited by the inclusion of studies that have been published in English only, we had not clearly stated this in its separate section after the abstract. However, we have rectified that and included a Strengths and Limitations section.

Overall Comment
This manuscript describes the findings of a systematic review to identify studies reporting patient-important outcomes when assessing mRDT effectiveness. The authors suggest that effectiveness studies incorporate process evaluations to capture contextual and patient-related factors that may impact the design. The strengths of this manuscript include: The paper is well-organized and justified>> The methods are clear and could be repeated>> The tables, figures and supplements illustrate review findings, making them easy to understand. While the idea around this paper is good, I think the execution needs more work, which prevents a recommendation for it to move forward to publication at this time. Below, I list major issues that should be addressed, and a number of minor issues which could be considered. Agreed and thank you for your constructive feedback. We have incorporated all your comments as depicted above which we believe have helped clarify its execution.

Reviewer 2
Major Comments
The rationale behind including only studies published in English should be clearly justified. Given the geographic focus on sub-Saharan Africa, were relevant studies published in French or Portuguese excluded? This could limit generalizability. Agreed. We have rephrased the section on Strengths and Limitations to include the impact of including studies published in English only as follows: “Although we only included studies published in English which could affect generalizability of these findings, we believe this is a representative sample.” See pages 33-34.
Along similar lines, the studies found seem to come from a limited number of countries. Do you think these results can be generalized to all sub-Saharan Africa? Agreed and thank you for noticing this. We expanded on this and discussed its impact on generalizability. See page 34.
Some further clarification on the definitions/categorization of patient-important outcomes would be helpful, or reference to a framework used. For example, are measures considered direct or indirect? Agreed. We have included a reference to the framework used. See page 7.
The quality assessment revealed some key limitations of primary studies included. It would be useful to discuss how this could have biased the results or influenced the validity of findings. Agreed. We have discussed how the key limitations identified from the quality assessment, could have impacted the validity of findings. See page 34.
The implementation concerns seem highly relevant but could be structured or themed in a more meaningful way. Is there an existing framework, the challenges could be mapped to? This would better highlight the most pressing issues. Agreed and thank you.
We used an existing framework: Supporting the Use of Research Evidence (SURE) to map out the implementation challenges that were identified from the included studies. See page 9.
The implications for policy, practice and research could be expanded. In particular, more details on how the methodology of future mRDT impact studies could be improved would further add value. Agreed. We have included more details on the methodology of future mRDTs. See page 35.

Minor Comments
Explain acronyms before first use (e.g. NMCPs). Agreed and thank you; however, we had initially explained the full meaning of the acronym NMCPs as National Malaria Control Programs as in Page 4, line 19. We have also double-checked other acronyms used to find out any that might have been left out.

The objective should specify focus is on methodological evaluations. Agreed and thank you. We have rectified the section to read: “To perform critical methodological assessments on designs, outcomes, quality, and implementation limitations of studies evaluating the impact of mRDTs on patient-important outcomes in sub-Saharan Africa”. See page 2-5.

Overall Comment
This is an important systematic review evaluating the methodology and implementation challenges of studies assessing the impact of malaria rapid diagnostic tests (mRDTs) on patient-important outcomes in sub-Saharan Africa. The topic is highly relevant given the widespread use of mRDTs and need to understand their real-world impact. The manuscript is clearly written and provides helpful insights. I have outlined some suggestions below to further strengthen the manuscript. Overall, this is an insightful, methodologically focused systematic review. Addressing the suggestions above would further improve clarity and impact. Agreed and thank you for your constructive feedback. We have incorporated all your comments as depicted above.
